# Identification and functional implications of pseudouridine RNA modification on small noncoding RNAs in the mammalian pathogen *Trypanosoma brucei*

**DOI:** 10.1016/j.jbc.2022.102141

**Published:** 2022-06-14

**Authors:** K. Shanmugha Rajan, Katerina Adler, Tirza Doniger, Smadar Cohen-Chalamish, Noa Aharon-Hefetz, Saurav Aryal, Yitzhak Pilpel, Christian Tschudi, Ron Unger, Shulamit Michaeli

**Affiliations:** 1 The Mina and Everard Goodman Faculty of Life Sciences and Advanced and Nanotechnology Institute, Bar-Ilan University, Ramat-Gan, Israel; 2 Department of Molecular Genetics, Weizmann Institute of Science, Rehovot, Israel; 3 Department of Epidemiology and Microbial Diseases, Yale School of Public Health, New Haven, Connecticut, USA

**Keywords:** noncoding RNA, pseudouridine, 2′-*O*-methylation, 7SL RNA, vtRNA, snoRNA, tRNA, BSF, bloodstream form, cDNA, complementary DNA, CMC, N-Cyclohexyl-N′-(β-[N-methylmorpholino]ethyl)carbodiimide p-toluenesulfonate, ncRNA, noncoding RNA, Nm, 2′-*O*-methylation, PCF, procyclic form, PRS, postribosomal supernatant, PUS, pseudouridine synthase, RNP, ribonucleoprotein, RT, reverse transcription, SL RNA, spliced leader RNA, TGIRT, thermostable group II intron reverse transcriptase, tAI, tRNA adaptation index, vtRNA, vault RNA

## Abstract

*Trypanosoma brucei*, the parasite that causes sleeping sickness, cycles between an insect and a mammalian host. However, the effect of RNA modifications such as pseudouridinylation on its ability to survive in these two different host environments is unclear. Here, two genome-wide approaches were applied for mapping pseudouridinylation sites (Ψs) on small nucleolar RNA (snoRNA), 7SL RNA, vault RNA, and tRNAs from *T. brucei*. We show using HydraPsiSeq and RiboMeth-seq that the Ψ on C/D snoRNA guiding 2′-*O*-methylation increased the efficiency of the guided modification on its target, rRNA. We found differential levels of Ψs on these noncoding RNAs in the two life stages (insect host and mammalian host) of the parasite. Furthermore, tRNA isoform abundance and Ψ modifications were characterized in these two life stages demonstrating stage-specific regulation. We conclude that the differential Ψ modifications identified here may contribute to modulating the function of noncoding RNAs involved in rRNA processing, rRNA modification, protein synthesis, and protein translocation during cycling of the parasite between its two hosts.

2′-*O*-methylation (Nm) and pseudouridinylation (Ψ) are the most abundant RNA modifications in eukaryotes (1, 2). These modifications are guided either by C/D or H/ACA small nucleolar RNAs (snoRNAs) or methyltransferase/pseudouridine synthase (PUS) enzymes, which guide Nm and Ψ, respectively (1, 2, 3, 4). Ψ contributes to structural stability and increased stacking interactions of the RNA due to the extra hydrogen bond formation compared to uridine (5). The guiding rules of Ψ modification require noncontinuous bipartite complementarity to the target site located 14 to 16 nt from the H/ACA box (2, 3). RNA modifications on spliceosomal U1, U2, U4, and U5 small nuclear RNAs (snRNAs) as opposed to rRNA modifications that take place in the nucleolus are carried out in the *Cajal* bodies by small *Cajal* body associated RNA species (6). Most snRNA modifications are constitutive, but Ψs could also be induced under stress on U2 snRNA, compromising splicing (7). Ψ modifications on snRNAs were also shown to direct cancer-associated splicing (8).

*Trypanosoma brucei* is the causative agent of sleeping sickness. The parasite cycles between two hosts, which require major adaptation to changes in temperature and nutrient milieu, among other conditions. In the tsetse fly, the stage known as procyclic form (PCF) propagates in the midgut and undergoes transformation to a metacyclic stage; these parasites are transferred to the mammalian host upon feeding by the infected fly. In the mammalian host, trypanosomes replicate in the blood as the bloodstream form (BSF) (9). Trypanosomes harbor unique RNA processing pathways, such as *trans-*splicing (10) and RNA editing (11), but lack transcriptional regulation of protein coding genes, and gene expression is mainly regulated by mRNA stability and translation (12). In *trans-*splicing, the small RNA known as spliced leader RNA (SL RNA) donates a 5′ exon to all mRNAs (13, 14). The SL RNA is transcribed from a RNA polymerase-II promoter (15) and undergoes very unique modification to form a complex cap4 structure composed of m^7^G and four hypermodified nt (16). The SL RNA also undergoes pseudouridylation by the H/ACA snoRNA known as spliced leader–associated RNA (SLA1/TB11Cs2H1) (17). The main function of SLA1 is not in directly guiding the modification on Ψ28 but its chaperone-like activity during the early biogenesis of SL RNA to maintain a structure that is most suitable for cap 4 modification (18).

Trypanosomes possess a rich repertoire of snoRNAs comprising 83 H/ACA snoRNAs and 85 C/D snoRNAs (19, 20, 21). Trypanosome H/ACA snoRNAs are unique as they are composed of a single hairpin compared to a double hairpin structure in most other eukaryotes and possess an AGA instead of an ACA box (22). Ψ-seq on rRNA isolated from both life cycle stages identified 68 Ψs, including several developmentally regulated sites, and overexpression of snoRNAs guiding hypermodified sites revealed their role in adaptation, while cycling between the two hosts (21). Most striking was the finding that *T. brucei* U1, U2, U4, U5, and U6 snRNAs possess ∼61 Ψs that are mostly guided by snoRNAs (23). The *T. brucei* snoRNAs that guide snRNA Ψ modification bind to the same protein that was shown to bind SLA1 RNA, known as methyltransferase-associated protein (MTAP) (23). *mtap* silencing eliminated Ψs on snRNAs though not on rRNA, but silencing of *cbf5*, the PUS associated with snoRNAs, affected both rRNA and snRNA Ψ modification, suggesting that many trypanosome H/ACAs guide modifications on both rRNA and snRNA (23). Ψ modifications on snRNAs strengthen RNA–RNA and RNA–protein interactions at elevated temperatures (23). Over 250 Ψs were also mapped on *T. brucei* mRNAs by Ψ-seq, and several of those were shown to be guided by PUS enzymes. Interestingly, Ψ modification on mRNA 3′UTRs inhibit association of mRNA-binding proteins with their target mRNA (24).

Several trypanosome noncoding RNAs (ncRNAs) have unique properties compared to other eukaryotes. As an example, Trypanosomes possess a homolog of 7SL RNA, but in contrast to all eukaryotes, the signal recognition particle is composed of two RNA molecules: the 7SL RNA and a tRNA-like molecule, sRNA-76 (25, 26). The tRNA-like molecule interacts by base pairing with the Alu-like domain at the 5′ end of the 7SL RNA (27). In addition, vault RNA (vtRNA) was recently shown to exist in trypanosomes and to possess unique functions. Downregulating vtRNA levels impaired *trans*-splicing and mRNA production, suggesting its role in mRNA metabolism (28).

Very little is known regarding the Ψ modification of ncRNAs other than that of the abundant snRNAs (29). It was previously suggested based on primer extension mapping that in mammals, vault RNA carries 4 Nm sites and 7SL RNA contains five Ψs (30). In this study, we utilized both small RNA Ψ-seq (23) and HydraPsiSeq (31) to map Ψs on SL RNA, 7SL RNA, vtRNA, C/D and H/ACA snoRNAs, and tRNAs. Differential levels of Ψ modification were observed between the BSF and PCF stages. The presence of Ψ on C/D snoRNA affected its ability to guide Nm modification, since in *cbf5* silenced cells, the Nm guided by C/D snoRNA was significantly reduced. tRNA levels and Ψ modification were also shown to differ between the BSF and PCF parasites. This study highlights the potential role of Ψ modifications on ncRNAs for gene regulation at different developmental stages.

## Results

### Genome-wide mapping of Ψs on small ncRNA isolated from postribosomal supernatant

Previously we used more than 14 independent biological replicates of small RNA Ψ-seq libraries to detect Ψs mostly on snRNAs involved in splicing (23). Here, we used the same libraries to identify Ψs on additional classes of abundant ncRNAs. To validate the detected Ψs by reverse transcription (RT)-independent mapping as well as to quantify the stoichiometry of these novel Ψs, we sought to establish a modified protocol adjusted for small RNAs using HydraPsiSeq (31). Ψ-seq relies on the ability of reverse transcriptase to terminate 1 nt before the actual Ψ residue when encountering the “bulky” N-Cyclohexyl-N′-(β-[N-methylmorpholino]ethyl)carbodiimide p-toluenesulfonate (CMC) modified Ψ site (23, 24). HydraPsiSeq is based on random cleavage of only unmodified uridine nucleotides in RNA by utilizing the combination of hydrazine and aniline treatment and sequencing of fragmented RNA containing intact Ψ-modified residues (31).

**Figure 1:**
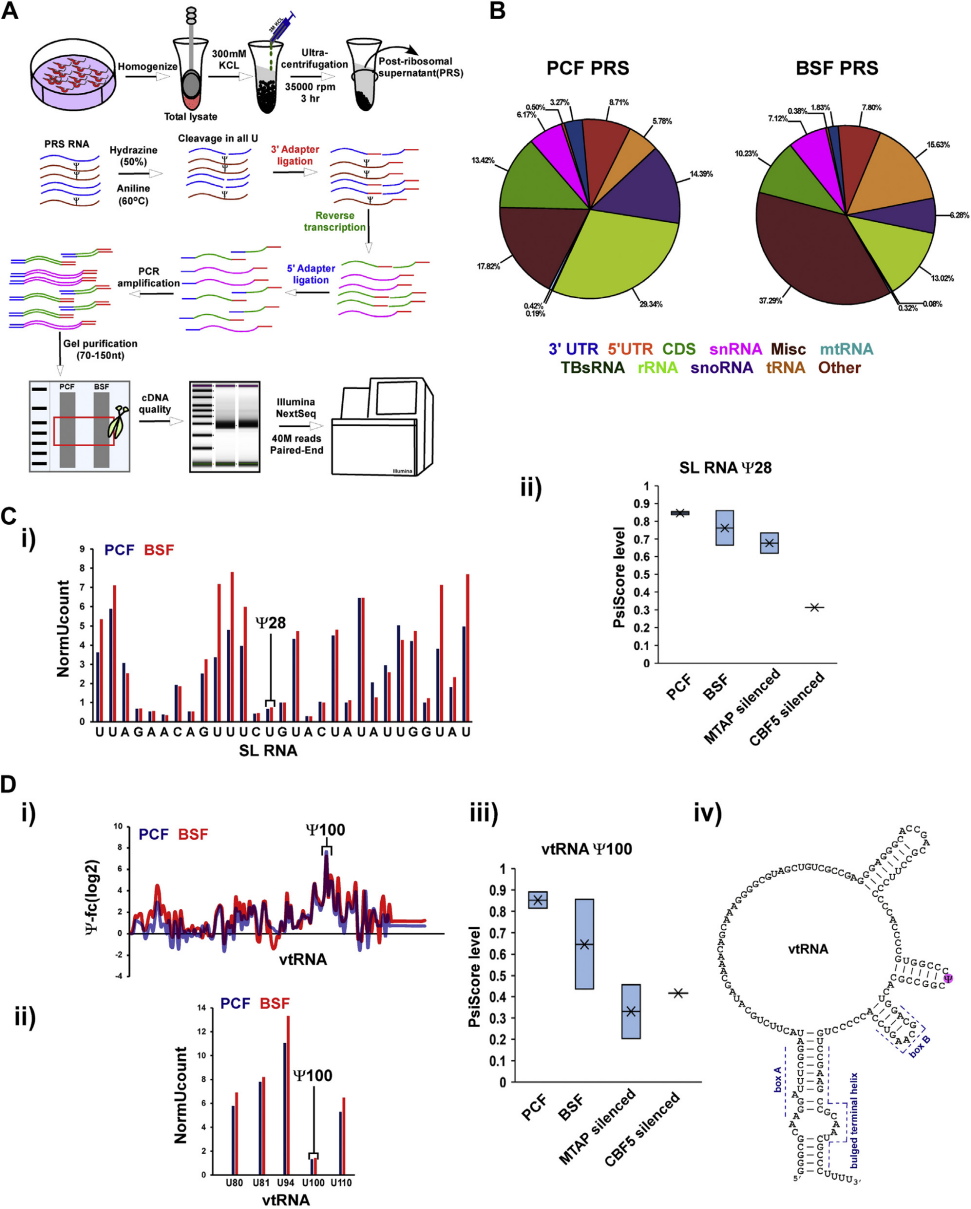
**Genome-wide small RNA HydraPsiSeq in *T. brucei*.***A*, schematic presentation of the small RNA HydraPsiSeq protocol. The scheme illustrates the extract preparation, fractionation, and the small RNA HydraPsiSeq methodology. *B*, enrichment of small RNAs in small RNA HydraPsiSeq libraries. Whole-cell extracts from 5 × 10^9^ PCF and BSF cells were prepared, depleted of ribosomes, RNA was extracted and subjected to hydrazine-aniline treatment, and used to prepare small RNA libraries, as described in (*A*). Graphs show proportion of ncRNA in the libraries. *C*, validation of HydraPsiSeq libraries using known Ψ28 in SL RNA. (i) Fragmentation profile of SL RNA in small RNA HydraPsiSeq libraries. The normalized uridine cleavage profile (NormUcount) was calculated as previously described (31). *Lower* NormUcount indicates the resistance to hydrazine-aniline treatment, demonstrating the presence of Ψ28 in SL RNA. (ii) Stoichiometry of Ψ28 in SL RNA. RNA extracted from the indicated cell lines was subjected to hydrazine-aniline treatment, and HydraPsiSeq analysis was performed as described in (*A*). Two independent replicates were used for the analysis. Data are presented as *box plot*. *D*, detection of novel Ψ in vtRNA. (i) Small RNA Ψ-seq detected a single Ψ on vtRNA. A representative *line graph* of the Ψ-fc(log2) (+CMC/-CMC) is presented for vtRNA from PCF and BSF RNAs. The results indicate a single Ψ100 in both the life stages. (ii) Fragmentation profile of vtRNA in small RNA HydraPsiSeq libraries. *Lower* NormUcount indicating the resistance to hydrazine-aniline treatment validating the presence of Ψ100 in vtRNA. (iii) Stoichiometry of Ψ100 in vtRNA. RNA extracted from the indicated cell lines was subjected to hydrazine-aniline treatment, and HydraPsiSeq analysis was performed as described in (*A*). The fraction of pseudouridine (PsiScore) was calculated as previously described (31). Two independent replicates were used for the analysis, except for *cbf5* +TET, which was tested in a single replicate. Data are presented as *box plot*. (iv) Scheme depicting the position of Ψ100 on the secondary structure of vtRNAs as previously determined by us (28). BSF, bloodstream form; CMC, N-Cyclohexyl-N′-(β-[N-methylmorpholino]ethyl)carbodiimide p-toluenesulfonate; PCF, procyclic form; SL RNA, spliced leader RNA; vtRNA, vault RNA.

To enrich for small ncRNAs, whole-cell extracts were prepared from both PCF and BSF *T. brucei* parasites, depleted of ribosomes by ultracentrifugation, and the resulting postribosomal supernatant (PRS) was utilized to extract fractions enriched in small ncRNA (Fig. 1*A*) (23, 32). To map Ψ residues, the RNA prepared from PRS was subjected to hydrazine-aniline treatment, and the fragmented RNAs were subjected to deep sequencing, obtaining more than 40 million reads for each library. Small RNA HydraPsiSeq libraries were prepared from the two life stages of the parasite. In these libraries, rRNA was reduced significantly to less than 14% to 30% of the total preparation, compared to 80%-90% in total RNA (Fig. 1*B*). To enable quality assessment of PCF and BSF small RNA HydraPsiSeq libraries, the fragmentation profile of the Ψ on SL RNA was determined. SL RNA has a single Ψ28 guided by SLA1 RNA (17). The data (Fig. 1*C*i) suggested that all uridine residues along the SL RNA were fragmented (as measured by the NormUcount score (31)) except for Ψ28, confirming that Ψ residues are resistant to cleavage.

To determine the fraction of pseudouridylation on each uridine residue, the PsiScore was calculated, which takes into consideration the total number of reads and the 5’/3′ end coverage in each uridine nucleotide (31). Next, we quantified the stoichiometry of Ψ28 on SL RNA derived from both life stages. Our results (Fig. 1*C*ii) indicated that Ψ28 is fully modified in both life stages of the parasite. To examine the ability of the method to detect variations in Ψ level, we also mapped Ψ on RNA derived from cells silenced for *mtap* and *cbf5*. MTAP is a protein that was shown to bind to SLA1 (18) as well as to H/ACA snoRNA guiding on both rRNA and snRNA (18), whereas CBF5 is the PUS associated with all H/ACA snoRNA (33). Our results (Fig. 1*C*ii) verified the dependance of Ψ28 on these two proteins, as previously described (18, 23).

Next, we analyzed the Ψs on vtRNA (28). A single site was located at position Ψ100 by both small RNA Ψ-seq (Fig. 1*D*i) and HydraPsiSeq (Fig. 1*D*ii-iii), and the Ψ was plotted on a scheme showing the experimentally verified secondary structure of vtRNA (Fig. 1*D*iv). This position is modified to a similar degree on both life stages (Fig. 1*D*ii) and is guided by dual functioning snoRNA, as based on small RNA Ψ-seq (Fig. S1) and HydraPsiSeq (Fig. 1*D*iii), the stoichiometry of Ψ100 is reduced upon *cbf5* and *mtap* silencing.

### Ψs on 7SL RNA and U3 snoRNA are developmentally regulated

**Figure 2:**
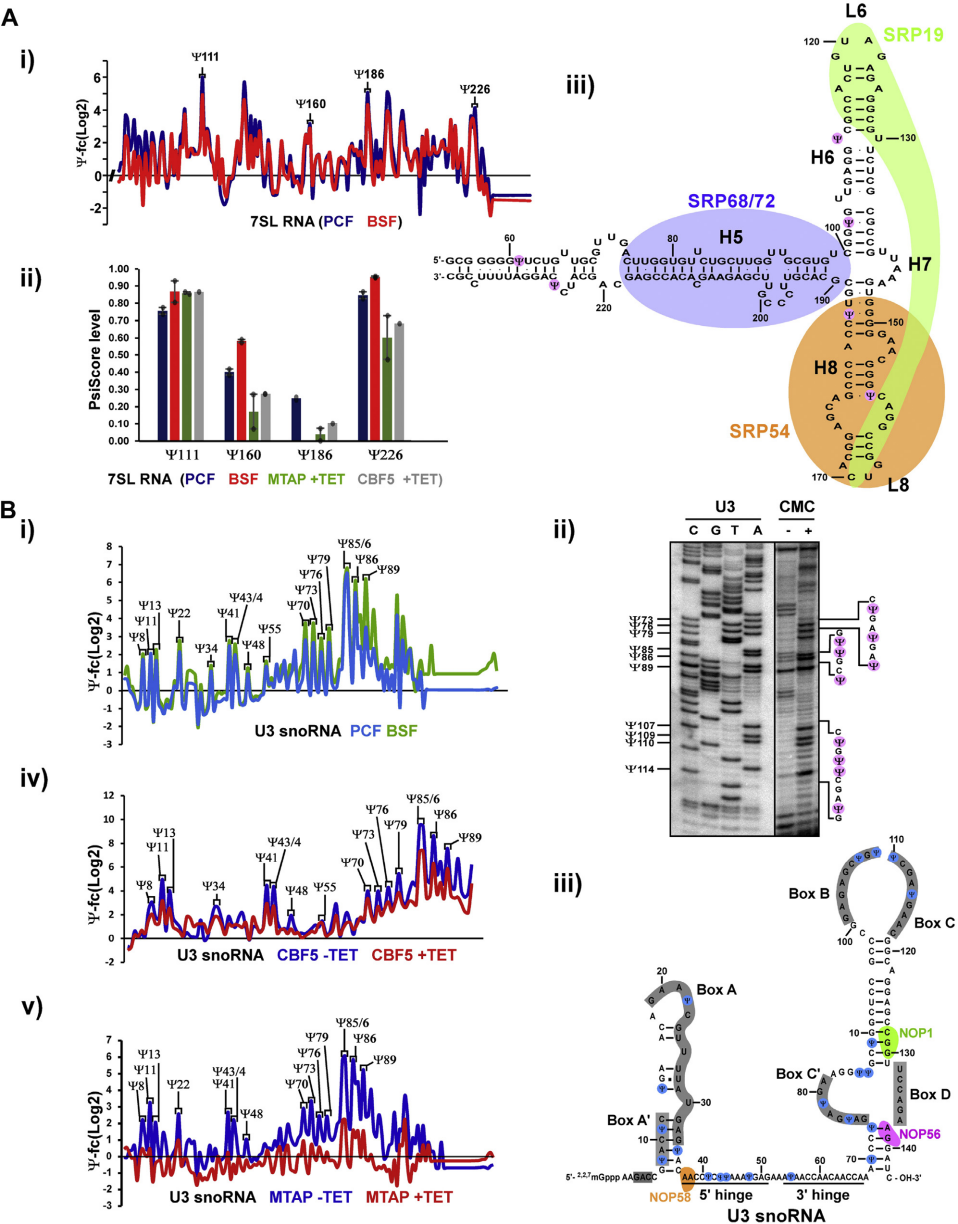
**Detection of novel Ψ in *T. brucei* 7SL and U3 RNA.***A*, Ψs on 7SL RNA. (i) Small RNA Ψ-seq detected a several Ψ on 7SL RNA. A representative *line graph* of the Ψ-fc(log2) (+CMC/-CMC) is presented for the 3′ region of 7SL RNA from PCF and BSF RNAs. The results indicate the presence of four Ψs in this region in both the life stages. (ii) Stoichiometry of Ψs on 7SL RNA. RNA extracted from the indicated cell lines were subjected to hydrazine-aniline treatment, and HydraPsiSeq analysis was performed. The fraction of pseudouridine (PsiScore) was calculated as previously described (31). Two independent replicates were used for the analysis, except for CBF5 +TET, which was analyzed in a single replicate. Data are presented as mean ± SD. (iii) Ψs are enriched in RNA–RNA and RNA–protein interaction domains of 7SL RNA. Scheme depicting the position of *T. brucei* Ψs on the secondary structure of 7SL RNAs based on small RNA Ψ-seq and HydraPsiSeq, highlighting functional domains; protein and RNA interaction regions are indicated (26). *B*, Ψs on U3 snoRNA. (i) Small RNA Ψ-seq detected multiple Ψs on U3 snoRNA. A representative *line graph* of the Ψ-fc(log2) (+CMC/-CMC) is presented for U3 from PCF and BSF RNAs. (ii) Validation of Ψs in *T. brucei* U3 snoRNA. Total RNA (100 μg) treated with CMC (+CMC) or untreated (-CMC) was subjected to primer extension with region-specific primers and analyzed on a 12% polyacrylamide gel (7M urea). The results alongside DNA sequencing performed using the same primer are presented. The positions of the Ψs are indicated (1 nt after the actual stop seen in the gel), as well as the RNA sequence. Contrast adjusted blots are separated by *bold lines*. (iii) Ψs are enriched in RNA–RNA and RNA–protein interaction domains of U3 snoRNA. Scheme depicting the position of *T. brucei* Ψs on the secondary structure of 7SL RNAs based on small RNA Ψ-seq and primer extension analysis, highlighting functional domains; protein and RNA interaction regions are indicated (35, 47). (iv) Ψs on U3 snoRNA are guided by H/ACA snoRNA. Ψ-fc(log2) values (*y*-axis) were determined for both *cbf5* -TET and +TET based on small RNA Ψ-seq libraries. Representative line graph of U3 is presented. Three independent biological replicates of small RNA Ψ-seq were used to validate the dependence of *T. brucei* U3 Ψs on *cbf5* silencing. (v) Ψs on U3 snoRNA are guided by H/ACA snoRNA associated with MTAP. Ψ-fc(log2) values (*y*-axis) were determined for both *mtap* -TET and +TET based on small RNA Ψ-seq libraries. Representative line graph of U3 is presented. Three independent biological replicates of small RNA Ψ-seq were used to validate the dependence of *T. brucei* U3 Ψs on *mtap* silencing. BSF, bloodstream form; CMC, N-Cyclohexyl-N′-(β-[N-methylmorpholino]ethyl)carbodiimide p-toluenesulfonate; PCF, procyclic form; SL RNA, spliced leader RNA.

Using small RNA Ψ-seq with stringent criteria (log2 (Ψ-fc) of >3 and Ψ-ratio of > 0.01) and based on 14 libraries, 11 putative Ψ sites were detected on 7SL RNA (Fig. 2*A*i) with nine sites verified by HydraPsiSeq (Table 1 and Fig. 2*A*ii). Note that our HydraPsiSeq analysis could not detect Ψ on uridine residues carrying both Ψ and 2′-*O*-methylation sites in *T. brucei* rRNA, for an unknown reason. Next, we investigated the localization of these Ψs on the secondary structure depicting the conserved domains of the *T. brucei* 7SL RNA (Fig. 2*A*iii) (26). Note that only six Ψs are depicted in Figure 2*A*iii and the remaining three verified Ψ sites are located at the 5′ end domain with unknown structure (26) and thus were not presented. Three of the depicted Ψs sites (Ψ103, 160, and 186) were located within G-U base pairing regions, and one (Ψ61) was detected close to a G-U base pair. Two Ψ sites (Ψ160 and 186) were found in the domain interacting with the signal peptide-binding protein, SRP54. The sites at the 5′ end domain are of interest as they are likely to be involved in the interaction with the tRNA-like molecule present in the *T. brucei* SRP complex (25, 26, 27). Next, we verified the authenticity of these sites by determining their level following *cbf5* and *mtap* silencing, which we showed to reduce the level of Ψ on snRNAs (Fig. 2*A*ii) (23). Indeed, using the HydraPsiSeq data (described in Fig. 1), we observed reduction in the level of 7 Ψs under the silencing of both *cbf5* and *mtap*, suggesting that these modifications are most likely guided by dual functioning snoRNAs, in analogy to the snoRNA guiding on snRNAs (23) (Table 1). Interestingly, Ψ160 located in the SRP54-binding domain seems to be hypermodified in BSF, and Ψ186 was detected only in PCF (Table 1). To identify the snoRNAs guiding these Ψ positions on 7SL RNA, we used psoralen UV-induced crosslinking RNA libraries composed of chimeric RNA molecules ligated between the small RNA and its targets (Fig. S2, Tables S2 and S1) (23, 32). These libraries were used previously to identify the snoRNAs guiding Ψ on U2 snRNA, as well as the target of novel ncRNAs (23, 32). Six snoRNAs that obey the conventional guiding rules for Ψ on 7SL RNA were identified (Table 1, Fig. S2).

**Table 1:** Stoichiometry of Ψs on small ncRNAs determined by HydraPsiSeq

Ψ position	ncRNA	PCF (PsiScore)	BSF (PsiScore)	MTAP (PsiScore)	CBF5 (PsiScore)	Putative guide snoRNA
Ψ26	7SL RNA	0.90 ± 0.02	0.79 ± 0.05	0.76 ± 0.07	0.60	TB6Cs1H2
Ψ33	7SL RNA	0.72 ± 0.06	0.46 ± 0.05	0.51 ± 0.03	0.43	TB9Cs9H1
Ψ44	7SL RNA	0.74 ± 0.01	0.60 ± 0.02	0.68 ± 0.01	0.47	
Ψ61	7SL RNA	0.65 ± 0.01	0.41 ± 0.05	0.46 ± 0.05	0.57	
Ψ103	7SL RNA	0.17 ± 0.09	0.40 ± 0.13	0.33 ± 0.00	0.48	
Ψ111	7SL RNA	0.76 ± 0.02	0.87 ± 0.06	0.86 ± 0.01	0.87	TB7Cs3H2
Ψ160	7SL RNA	0.40 ± 0.02	0.58 ± 0.01	0.17 ± 0.10	0.28	TB11Cs3H2
Ψ186	7SL RNA	0.25 ± 0.01	0.00 ± 0.00	0.04 ± 0.04	0.10	TB9Cs1ppH1
Ψ226	7SL RNA	0.85 ± 0.02	0.95 ± 0.00	0.60 ± 0.13	0.68	TB10Cs2ppC4
Ψ28	SL RNA	0.85 ± 0.01	0.76 ± 0.10	0.68 ± 0.06	0.31	TB11Cs2H1
Ψ28	TB10Cs1C4	0.65 ± 0.04	0.24 ± 0.17	0.67 ± 0.04	0.47	TB10Cs1pH3/TB7Cs3H1/TB9Cs5H1
Ψ31	TB11Cs2C1	0.79 ± 0.01	0.07 ± 0.13	0.44 ± 0.13	0.77	
Ψ34	TB11Cs2C2	0.90 ± 0.01	0.79 ± 0.03	0.84 ± 0.04	0.72	
Ψ18	TB11Cs2H1	0.25 ± 0.03	0.38 ± 0.09	0.00 ± 0.00	0.35	TB10Cs1H3/TB10Cs3H2/TB10Cs1pH2
Ψ20	TB11Cs2H1	0.63 ± 0.06	0.65 ± 0.09	0.27 ± 0.31	0.12	
Ψ40	TB11Cs3C2	0.60 ± 0.12	0.54 ± 0.01	0.69 ± 0.08	0.44	
Ψ25	TB7Cs3H1	0.60 ± 0.03	0.33 ± 0.01	0.63 ± 0.02	0.00	TB11Cs3H2
Ψ26	TB7Cs3H1	0.69 ± 0.06	0.63 ± 0.00	0.75 ± 0.04	0.38	
Ψ58	TB7Cs3H2	0.34 ± 0.01	0.05 ± 0.36	0.60 ± 0.14	NA	
Ψ21	TB9Cs1ppH1	0.40 ± 0.01	0.57 ± 0.05	0.20 ± 0.05	NA	
Ψ28	TB9Cs1ppH1	0.92 ± 0.02	0.89 ± 0.00	0.90 ± 0.00	NA	TB10Cs-7H1
Ψ25	TB9Cs5C1	0.24 ± 0.09	0.36 ± 0.15	0.27 ± 0.11	0.00	
Ψ100	vtRNA	0.85 ± 0.04	0.65 ± 0.21	0.33 ± 0.13	0.42	TB10Cs4H4

The data are derived from at least two independent replicates, except for one replicate of *cbf5* silencing. Data are presented as mean ± SEM of PsiScore.

Next, we mapped all the Ψs on U3 (Fig. 2*B*i and ii). Note that because of the adjacent uridine residues on the snRNA, smaller fragments are generated during hydrazine-aniline cleavage, which are not suitable for sequencing in our experimental setup, and thus, HydraPsiSeq is not suitable for mapping Ψ on such U-rich small RNA molecules. Using small RNA Ψ-seq, we found that U3 snoRNA possess a rich repertoire of 22 Ψs. This is a surprising result since only four Ψs were mapped on human U3 (34). Among the 22 sites, three were reproducibly found to be hypermodified in the BSF compared to the PCF (Table S3). Mapping the Ψ sites on the secondary structure of U3 snoRNA showed that the Ψs are located in the stem-loop regions (Fig. 2*B*iii). Ψs were also located in loop domains such as Box A, A′, B, C, and C′, suggesting that as in U2, U4, and U6 snRNAs, Ψs in U3 are concentrated within RNA–protein interaction domains, as well as in RNA–RNA interaction domains that were shown to be crosslinked to pre-rRNA in the 5′ external transcribed spacer (35) (Fig. S3). Next, the dependence of these Ψs on snoRNAs was confirmed by their reduced level under *cbf5* and *mtap* silencing using Ψ-seq (Fig. 2*B*iv and v). Indeed all the detected Ψ sites were partially reduced in these silenced cell lines. Twelve snoRNAs were identified to potentially guide Ψ positions on U3 snoRNA by psoralen UV-induced crosslinking (23, 32) (Table S4).

### Ψs on snoRNAs and in the C/D interaction domain with rRNA affect Nm modification

**Figure 3:**
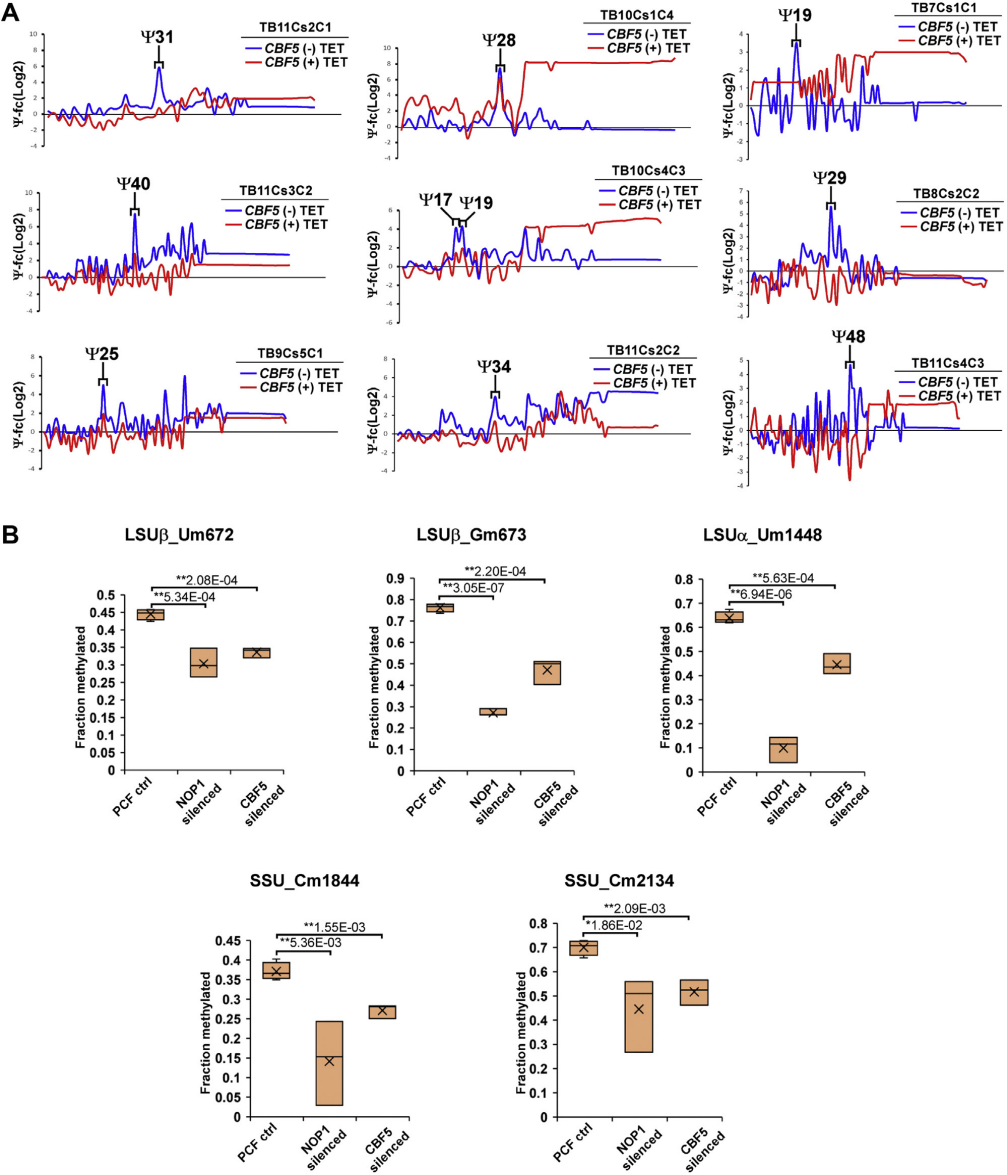
**Functional analysis of Ψs on *T. brucei* C/D snoRNAs.***A*, Ψs on C/D snoRNAs are guided by H/ACA snoRNA. Ψ-fc(log2) values (*y*-axis) were determined for both *cbf5* -TET and +TET based on small RNA Ψ-seq libraries. Representative line graph of six C/D snoRNAs is presented. Three independent biological replicates of small RNA Ψ-seq were used to validate the dependence of Ψs upon *cbf5* silencing. *B*, Ψ on *T. brucei* C/D snoRNA affect guided 2′-*O*-methylation on rRNA. The relative methylation score (ScoreC) representing the fraction of methylated nucleotides was calculated for individual Nm sites on rRNA as previously described (19, 39) and is plotted on the *y*-axis. *Lower score* indicates *lower level* of methylation on the site. At least three independent biological replicates were used to validate calculate the ScoreC. Data are presented as *box plot*. Student *t* test was used to calculate *p*-value. Student's *t* test was performed to determine the *p*-value. ∗*p* < 0.05, ∗∗<0.005.

Small RNA Ψ-seq and HydraPsiSeq libraries were also inspected for the presence of modifications on snoRNAs. By inspecting the small RNA Ψ-seq libraries, 10 Ψs were detected on 9 C/D snoRNAs (Fig. S4), with five Ψ sites verified by HydraPsiSeq (Table 1). Among these snoRNAs, five Ψs are present in abundant C/D snoRNAs that were shown to function in pre-rRNA processing (TB11Cs2C1, TB11Cs3C2, TB10Cs1C4, TB10Cs4C3, and TB11CS2C2) (36, 37). In all these cases, the snoRNA interacts by base pairing with intergenic regions of pre-rRNA but also within the rRNA sequence (36, 37). Ψs were found in the snoRNA–rRNA interaction domain for TB11Cs2C1, TB11Cs3C2, TB9Cs5C1, and TB10Cs1C4. However, the two Ψs detected in TB10Cs4C3 are located 3 to 5 nt from the predicted snoRNA–rRNA domain, which guides the corresponding Nm. TB11Cs2C2 and TB8Cs2C2 has a single Ψ detected next to the D′ box wherein an RNA-interaction domain guiding Nm could be plausible, but no such interaction domain has been predicted so far (19, 38). Moreover, this Ψ on TB11Cs2C2 is hypomodifed in BSF (Table 1). Similarly, TB7Cs1C1 and TB11Cs4C3 have one Ψ detected in a domain where no interaction domain with rRNA was predicted. The Ψs detected in TB11Cs2C2, TB8Cs2C2, TB7Cs1C1, and TB11Cs4C3 may suggest that these snoRNAs guide Nms on other classes of RNA, such as ncRNA or even mRNAs. Note, that all Ψs detected on C/D snoRNAs were reduced upon *cbf5* and/or *mtap* silencing (Fig. 3*A* and Table 1). The differential levels of the Ψs were all verified by HydraPsiSeq (Table 1).

To gain insight regarding the biological role of Ψ on C/D snoRNAs, we examined whether silencing of *cbf5*, which reduces the level of Ψ on the C/D snoRNAs, can affect the efficiency of guiding the cognate Nm modification on rRNA. To this end, the Nm modification guided by the C/D snoRNA was examined in *cbf5* silenced cells (Fig. 3*B*) by RiboMeth-seq (19, 39). In this Nm mapping method, the methylated nucleotide is resistant to alkaline hydrolysis and fragmentation profile is used to calculate the stoichiometry of the modified nucleotide (19, 39). The authenticity of these Nm modifications was verified in parallel by silencing C/D small nucleolar ribonucleoprotein (RNP) methyltransferase (NOP1). We identified five Nms where the levels are significantly reduced upon *cbf5* silencing; these five Nms are guided by four snoRNAs (TB7Cs1C1, TB8Cs2C2, TB10Cs4C3, and TB11Cs4C3) (Table 2 and Fig. 3*B*). This result indicates that the Nm modification guided by these four snoRNAs is strengthened by the Ψ, leading to more efficient methylation by the snoRNA. Note, in all these four snoRNAs, the Ψ is not located in region of base pairing with rRNA target, suggesting that these Ψ might affect the binding of C/D core RNPs as was previously suggested by us, demonstrating that Ψ strengthens the interaction of U2 snRNA with it binding proteins U2A′ and U2B” (23).

**Table 2:** Stoichiometry of Nm sites in *T. brucei* rRNA determined by RiboMeth-seq

Nm position	PCF control	CBF5 silenced	NOP1 silenced	FC (CBF5/PCF)	*p*-value (CBF5)	FC (NOP1/PCF)	*p*-value (NOP1)	snoRNA	nt
SSU_36	0.75 ± 0.02	0.74 ± 0.04	0.42 ± 0.23	0.98	0.6197	0.56	0.0338	TB10Cs2C1	U
SSU_46	0.57 ± 0.06	0.59 ± 0.04	0.60 ± 0.01	1.03	0.6458	1.05	0.4072	TB8Cs3C3	C
SSU_56	0.59 ± 0.04	0.54 ± 0.11	0.38 ± 0.13	0.91	0.3878	0.65	0.0244	TB8Cs2C1	A
SSU_66	0.34 ± 0.09	0.25 ± 0.05	0.32 ± 0.12	0.75	0.2150	0.94	0.8082	TB8Cs2C1	C
SSU_125	0.85 ± 0.02	0.85 ± 0.03	0.76 ± 0.08	1.00	0.9867	0.89	0.0553	TB8Cs1C2	A
SSU_680	0.48 ± 0.02	0.38 ± 0.20	0.40 ± 0.16	0.80	0.3753	0.83	0.3455	TB10Cs3C3	U
SSU_714	0.84 ± 0.02	0.81 ± 0.04	0.78 ± 0.03	0.97	0.3530	0.94	0.0427	TB9Cs2C6	U
SSU_721	0.80 ± 0.02	0.77 ± 0.05	0.72 ± 0.03	0.96	0.2331	0.90	0.0089	TB6Cs2C1	A
SSU_1517	0.49 ± 0.03	0.51 ± 0.10	0.29 ± 0.05	1.06	0.6113	0.58	0.0011	TB11Cs3C2	G
SSU_1531	0.81 ± 0.04	0.81 ± 0.07	0.43 ± 0.05	1.00	0.9562	0.53	0.0001	TB3Cs1C1	G
SSU_1603	0.91 ± 0.04	0.92 ± 0.03	0.86 ± 0.08	1.01	0.8237	0.94	0.3232	TB8Cs3C2	G
SSU_1630	0.42 ± 0.06	0.45 ± 0.11	0.16 ± 0.13	1.08	0.5848	0.39	0.0168	TB10Cs2"C3	U
SSU_1652	0.75 ± 0.01	0.75 ± 0.04	0.70 ± 0.12	0.99	0.7550	0.93	0.3887	TB10Cs1C3	U
SSU_1674	0.51 ± 0.03	0.52 ± 0.12	0.42 ± 0.23	1.01	0.9176	0.82	0.4411	TB10Cs3C2	U
SSU_1676	0.23 ± 0.08	0.46 ± 0.13	0.40 ± 0.18	2.03	0.0306	1.77	0.1303	TB9Cs3C1	G
SSU_1700	0.56 ± 0.03	0.49 ± 0.09	0.40 ± 0.04	0.87	0.1775	0.71	0.0015	TB10Cs2"C1	G
SSU_1844	0.37 ± 0.02	0.27 ± 0.02	0.14 ± 0.10	0.73	a0.0016	0.37	0.0054	TB7Cs1C1	C
SSU_1895	0.57 ± 0.07	0.57 ± 0.22	0.53 ± 0.17	1.01	0.9805	0.94	0.7130	TB9Cs2C4	G
SSU_1899	0.84 ± 0.03	0.83 ± 0.06	0.70 ± 0.13	0.99	0.7898	0.83	0.0693	TB11Cs2C1	U
SSU_2054	0.93 ± 0.00	0.86 ± 0.06	0.73 ± 0.02	0.93	0.0731	0.78	0.0000	TB8Cs2C0	U
SSU_2096	0.78 ± 0.01	0.82 ± 0.05	0.46 ± 0.12	1.05	0.1764	0.59	0.0030	TB9Cs3C2	A
SSU_2123	0.69 ± 0.04	0.57 ± 0.02	0.73 ± 0.13	0.84	0.0083	1.07	0.4979	TB10Cs4C3	U
SSU_2134	0.70 ± 0.03	0.52 ± 0.05	0.44 ± 0.15	0.74	a0.0021	0.63	0.0186	TB10Cs4C3	C
SSU_2154	0.25 ± 0.02	0.25 ± 0.05	0.18 ± 0.07	0.97	0.7700	0.69	0.0835	TB10Cs3C2	U
SSU_2227	0.88 ± 0.03	0.87 ± 0.06	0.83 ± 0.03	0.99	0.7461	0.95	0.1020	TB8Cs1C1	G
5.8S_41	0.79 ± 0.05	0.77 ± 0.08	0.53 ± 0.04	0.97	0.6858	0.67	0.0008	TB6Cs1′C1	A
5.8S_43	0.59 ± 0.07	0.53 ± 0.04	0.23 ± 0.20	0.91	0.3128	0.39	0.0197	TB6Cs1′C1	A
5.8S_75	0.44 ± 0.03	0.43 ± 0.06	0.25 ± 0.07	0.98	0.7761	0.56	0.0045	TB8Cs3C3	G
5.8S_163	0.94 ± 0.01	0.92 ± 0.07	0.50 ± 0.13	0.97	0.5255	0.53	0.0010	TB9Cs4C2	A
5.8S_167	0.89 ± 0.00	0.86 ± 0.06	0.83 ± 0.06	0.97	0.3619	0.93	0.1087	TB9Cs4C2	U
LSUa_254	0.34 ± 0.06	0.29 ± 0.13	0.19 ± 0.10	0.87	0.5740	0.57	0.0625	TB10Cs3C2	A
LSUa_742	0.59 ± 0.07	0.55 ± 0.14	0.30 ± 0.28	0.93	0.6159	0.50	0.0854	TB9Cs2C2	A
LSUa_743	0.92 ± 0.02	0.89 ± 0.05	0.59 ± 0.15	0.97	0.3115	0.64	0.0074	TB9Cs2C2	A
LSUa_746	0.92 ± 0.02	0.90 ± 0.05	0.86 ± 0.06	0.98	0.4973	0.93	0.0702	TB10Cs2C2	A
LSUa_747	0.69 ± 0.05	0.66 ± 0.08	0.49 ± 0.19	0.95	0.5327	0.71	0.0902	TB10Cs2C2	C
LSUa_760	0.82 ± 0.02	0.76 ± 0.05	0.61 ± 0.04	0.93	0.1144	0.75	0.0003	TB9Cs2C2	C
LSUa_916	0.73 ± 0.01	0.61 ± 0.08	0.47 ± 0.05	0.84	0.0307	0.65	0.0001	TB11Cs4C2	U
LSUa_925	0.84 ± 0.01	0.82 ± 0.04	0.80 ± 0.03	0.98	0.4063	0.96	0.0462	TB9Cs2C3	G
LSUa_927	0.75 ± 0.05	0.72 ± 0.05	0.67 ± 0.08	0.95	0.4279	0.89	0.1737	TB11Cs4C2	A
LSUa_996	0.57 ± 0.03	0.46 ± 0.12	0.11 ± 0.05	0.79	0.1071	0.19	0.0000	TB9Cs5C2	A
LSUa_1006	0.53 ± 0.05	0.51 ± 0.05	0.45 ± 0.01	0.96	0.5790	0.84	0.0335	TB9Cs5C1	C
LSUa_1024	0.81 ± 0.01	0.77 ± 0.05	0.46 ± 0.06	0.95	0.2079	0.57	0.0001	TB11Cs1C3	A
LSUa_1028	0.47 ± 0.05	0.36 ± 0.12	0.18 ± 0.13	0.77	0.1398	0.39	0.0099	TB9Cs2C5	G
LSUa_1145	0.79 ± 0.03	0.74 ± 0.13	0.72 ± 0.13	0.93	0.4552	0.91	0.3347	TB10Cs3C4	U
LSUa_1180	0.10 ± 0.10	0.15 ± 0.16	0.10 ± 0.18	1.47	0.6583	1.02	0.9791	TB9Cs5C1	A
LSUa_1181	0.91 ± 0.03	0.86 ± 0.09	0.64 ± 0.15	0.94	0.3201	0.70	0.0135	TB10Cs3C4	U
LSUa_1267	0.65 ± 0.02	0.66 ± 0.09	0.41 ± 0.17	1.02	0.7498	0.63	0.0319	TB9Cs4C1	G
LSUa_1448	0.64 ± 0.02	0.45 ± 0.04	0.09 ± 0.05	0.70	a0.0006	0.15	0.0000	TB8Cs2C2	U
LSUa_1605	0.73 ± 0.02	0.65 ± 0.14	0.65 ± 0.04	0.88	0.2610	0.88	0.0150	TB10Cs2"C3	G
LSUa_1608	0.55 ± 0.03	0.56 ± 0.06	0.22 ± 0.09	1.03	0.6503	0.40	0.0011	TB8Cs3C1	C
LSUa_1620	0.79 ± 0.06	0.74 ± 0.06	0.35 ± 0.02	0.94	0.3296	0.45	0.0001	TB8Cs3C1	A
LSUa_1621	0.50 ± 0.11	0.37 ± 0.07	0.38 ± 0.07	0.75	0.1555	0.76	0.1660	TB8Cs3C1	G
LSUa_1665	0.74 ± 0.01	0.67 ± 0.14	0.48 ± 0.01	0.91	0.3536	0.65	0.0000	TB6Cs1′C1	A
LSUa_1709	0.81 ± 0.03	0.80 ± 0.04	0.47 ± 0.05	0.99	0.7761	0.58	0.0001	TB8Cs1C1	G
LSUa_1742	0.83 ± 0.02	0.78 ± 0.10	0.81 ± 0.01	0.94	0.3885	0.97	0.2186	TB7Cs2C1	U
LSUb_71	0.52 ± 0.05	0.51 ± 0.06	0.34 ± 0.03	0.99	0.9409	0.67	0.0037	TB9Cs1C1	G
LSUb_73	0.69 ± 0.06	0.61 ± 0.04	0.28 ± 0.16	0.89	0.1368	0.41	0.0059	TB9Cs1C1	U
LSUb_95	0.76 ± 0.03	0.71 ± 0.12	0.39 ± 0.07	0.94	0.4549	0.52	0.0002	TB3Cs3C1	A
LSUb_377	0.49 ± 0.09	0.36 ± 0.14	0.02 ± 0.03	0.75	0.2138	0.04	0.0004	TB9Cs4C3	C
LSUb_400	0.93 ± 0.00	0.90 ± 0.06	0.73 ± 0.04	0.96	0.2913	0.78	0.0002	TB6Cs1C2	A
LSUb_520	0.78 ± 0.01	0.72 ± 0.10	0.65 ± 0.04	0.92	0.2548	0.84	0.0019	TB10Cs7C2	A
LSUb_544	0.20 ± 0.10	0.16 ± 0.05	0.05 ± 0.09	0.80	0.5461	0.25	0.0814	TB6Cs1C3	A
LSUb_545	0.79 ± 0.03	0.72 ± 0.12	0.47 ± 0.06	0.91	0.2907	0.59	0.0002	TB6Cs1C3	A
LSUb_552	0.70 ± 0.05	0.70 ± 0.06	0.48 ± 0.19	1.00	0.9394	0.68	0.0625	TB10Cs1C4	G
LSUb_578	0.91 ± 0.04	0.89 ± 0.04	0.76 ± 0.08	0.98	0.5643	0.84	0.0203	TB11Cs3C1	U
LSUb_588	0.48 ± 0.12	0.34 ± 0.30	0.10 ± 0.18	0.71	0.4210	0.21	0.0189	TB8Cs1C3	A
LSUb_601	0.53 ± 0.06	0.47 ± 0.03	0.42 ± 0.00	0.89	0.2075	0.78	0.0321	TB10Cs1C1	C
LSUb_609	0.58 ± 0.02	0.60 ± 0.02	0.47 ± 0.03	1.03	0.2617	0.81	0.0035	TB10Cs1C1	A
LSUb_622	0.79 ± 0.03	0.81 ± 0.08	0.62 ± 0.07	1.02	0.7209	0.78	0.0070	TB10Cs2′C1	A
LSUb_646	0.67 ± 0.01	0.50 ± 0.09	0.15 ± 0.08	0.75	0.0120	0.22	0.0000	TB11Cs4′C1	A
LSUb_659	0.26 ± 0.07	0.25 ± 0.15	0.48 ± 0.16	0.98	0.9467	1.86	0.0552	TB9Cs2C7	G
LSUb_672	0.44 ± 0.02	0.34 ± 0.01	0.30 ± 0.03	0.76	a0.0002	0.67	0.0005	TB11Cs4C3	U
LSUb_673	0.76 ± 0.02	0.47 ± 0.06	0.27 ± 0.02	0.62	a0.0002	0.36	0.0000	TB11Cs4C3	G
LSUb_685	0.64 ± 0.02	0.59 ± 0.05	0.57 ± 0.11	0.91	0.0904	0.88	0.2316	TB9Cs2C7	U
LSUb_728	0.24 ± 0.08	0.25 ± 0.11	0.09 ± 0.12	1.03	0.9165	0.37	0.0997	TB10Cs3C5	U
LSUb_1093	0.74 ± 0.03	0.60 ± 0.11	0.30 ± 0.05	0.81	0.0503	0.41	0.0000	TB5Cs1C1	U
LSUb_1094	0.61 ± 0.04	0.56 ± 0.10	0.38 ± 0.07	0.92	0.3969	0.63	0.0028	TB5Cs1C1	G
LSUb_1175	0.42 ± 0.05	0.36 ± 0.14	0.31 ± 0.18	0.85	0.4365	0.74	0.2960	TB5Cs1C1	C
LSUb_1201	0.77 ± 0.01	0.75 ± 0.04	0.42 ± 0.06	0.97	0.3078	0.55	0.0001	TB10Cs2"C2	A
LSUb_1245	0.72 ± 0.07	0.68 ± 0.08	0.63 ± 0.10	0.93	0.4311	0.86	0.1767	TB3Cs1C-1	G
LSUb_1247	0.76 ± 0.04	0.76 ± 0.05	0.44 ± 0.09	1.00	0.9986	0.58	0.0015	TB11Cs1C2	G
LSUb_1264	0.78 ± 0.03	0.67 ± 0.05	0.66 ± 0.07	0.86	0.0102	0.85	0.0240	TB6Cs1C1	C
LSUb_1269	0.43 ± 0.11	0.29 ± 0.16	0.50 ± 0.03	0.68	0.2265	1.16	0.3194	TB11Cs1C2	G
LSUb_1375	0.46 ± 0.07	0.51 ± 0.11	0.43 ± 0.16	1.09	0.5421	0.93	0.7205	TB10Cs3C1	U
LSUb_1388	0.42 ± 0.02	0.27 ± 0.12	0.23 ± 0.21	0.64	0.0435	0.55	0.1195	TB11Cs4C1	A
LSUb_1400	0.77 ± 0.03	0.57 ± 0.13	0.77 ± 0.08	0.74	0.0281	1.00	0.9708	TB11Cs4C1	A
LSUb_1413	0.80 ± 0.02	0.71 ± 0.11	0.78 ± 0.04	0.89	0.1793	0.99	0.6033	TB9Cs2C1	C
LSUb_1435	0.73 ± 0.03	0.65 ± 0.08	0.33 ± 0.01	0.88	0.1012	0.45	0.0000	TB9Cs3C3	U

The data are derived from at least three independent replicates. Data are presented as mean ± SEM of ScoreC (relative methylation score, RMS). The fold change of RMS score, *p*-value, and identity of snoRNA guiding individual Nms are also presented.

a *p* < 0.005.

**Figure 4:**
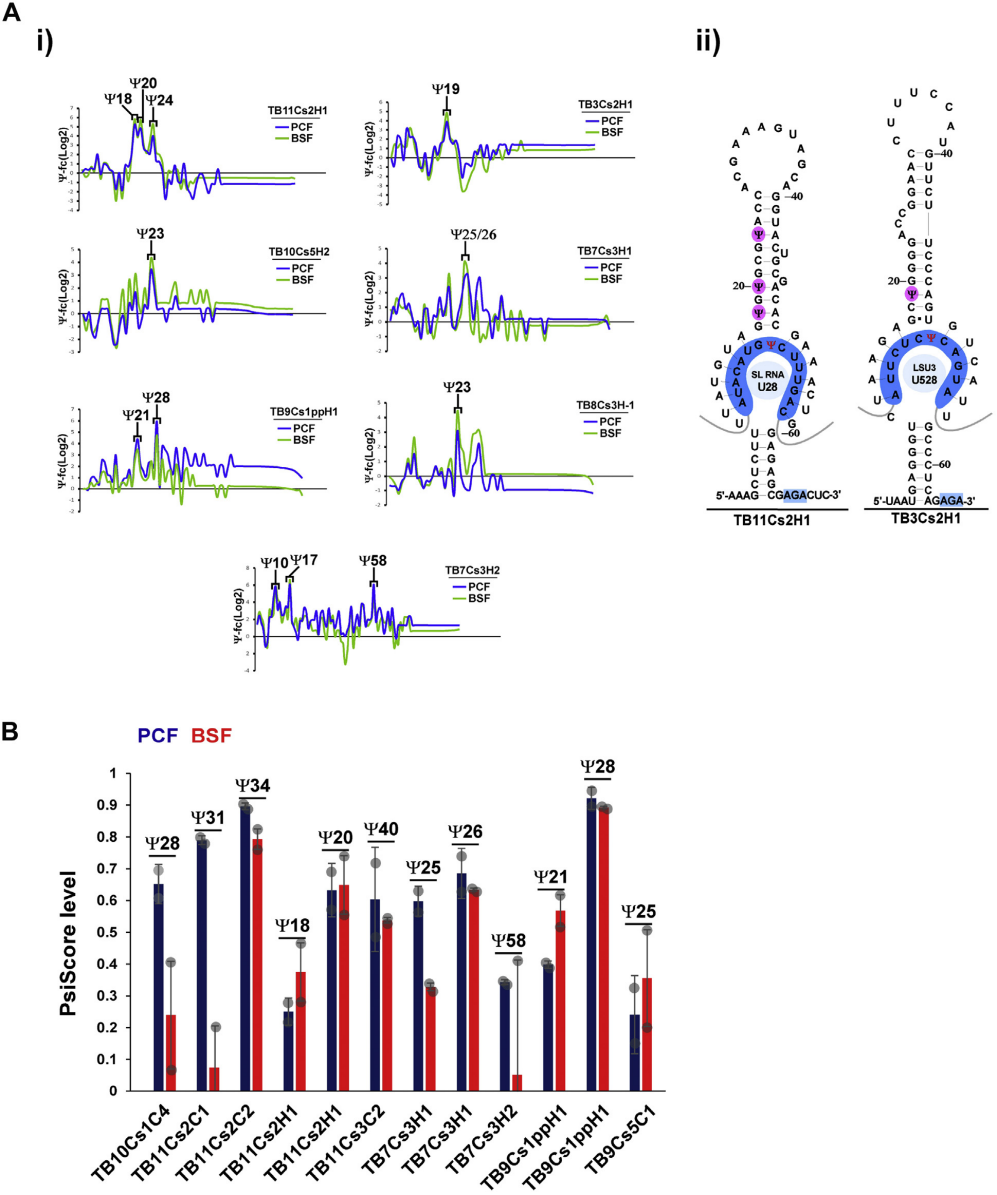
**Detection and quantification of Ψs on *T. brucei* snoRNAs.***A*, Ψs on *T. brucei* H/ACA snoRNAs. (i) Detection of novel Ψs on H/ACA snoRNAs by small RNA Ψ-seq. Ψ-fc(log2) values (*y*-axis) were determined from both PCF and BSF small RNA Ψ-seq libraries. Representative *line graphs* of seven H/ACA snoRNAs are presented. (ii) Localization of Ψs on *T. brucei* H/ACA snoRNA. Scheme depicting the position of Ψs on the secondary structure of H/ACA snoRNA, highlighting the pseudouridylation pocket; the potential base pairing with the rRNA target is indicated. *B*, stoichiometry of Ψs on snoRNAs. PRS RNA extracted from PCF and BSF parasites were subjected to hydrazine-aniline treatment, and the PsiScore was calculated. Two independent replicates were used for the analysis. Data are presented as mean ± SEM. BSF, bloodstream form; PCF, procyclic form.

Inspecting the small RNA Ψ-seq libraries as described previously, 13 Ψs were detected on seven H/ACA snoRNAs (Fig. 4*A*i) with seven Ψ sites verified by small RNA HydraPsiSeq (Table 1). Mapping the Ψs on the secondary structure scheme shows that most of these Ψs are present in the stem region above the pseudouridylation pocket (Fig. 4*A*ii). The level of the Ψs on H/ACA snoRNA was examined by HydraPsiSeq in the two life stages, and we found cases of both hypermodification and hypomodification of these sites in BSF (Fig. 4*B* and Table 1). The snoRNAs guiding Ψ positions on snoRNAs were identified and potential base pairing obeys the conventional guiding rules (Table 1 and Fig. S2).

### The levels of tRNAs are developmentally regulated and are coordinated with the level of mRNA in the two life stages

**Figure 5:**
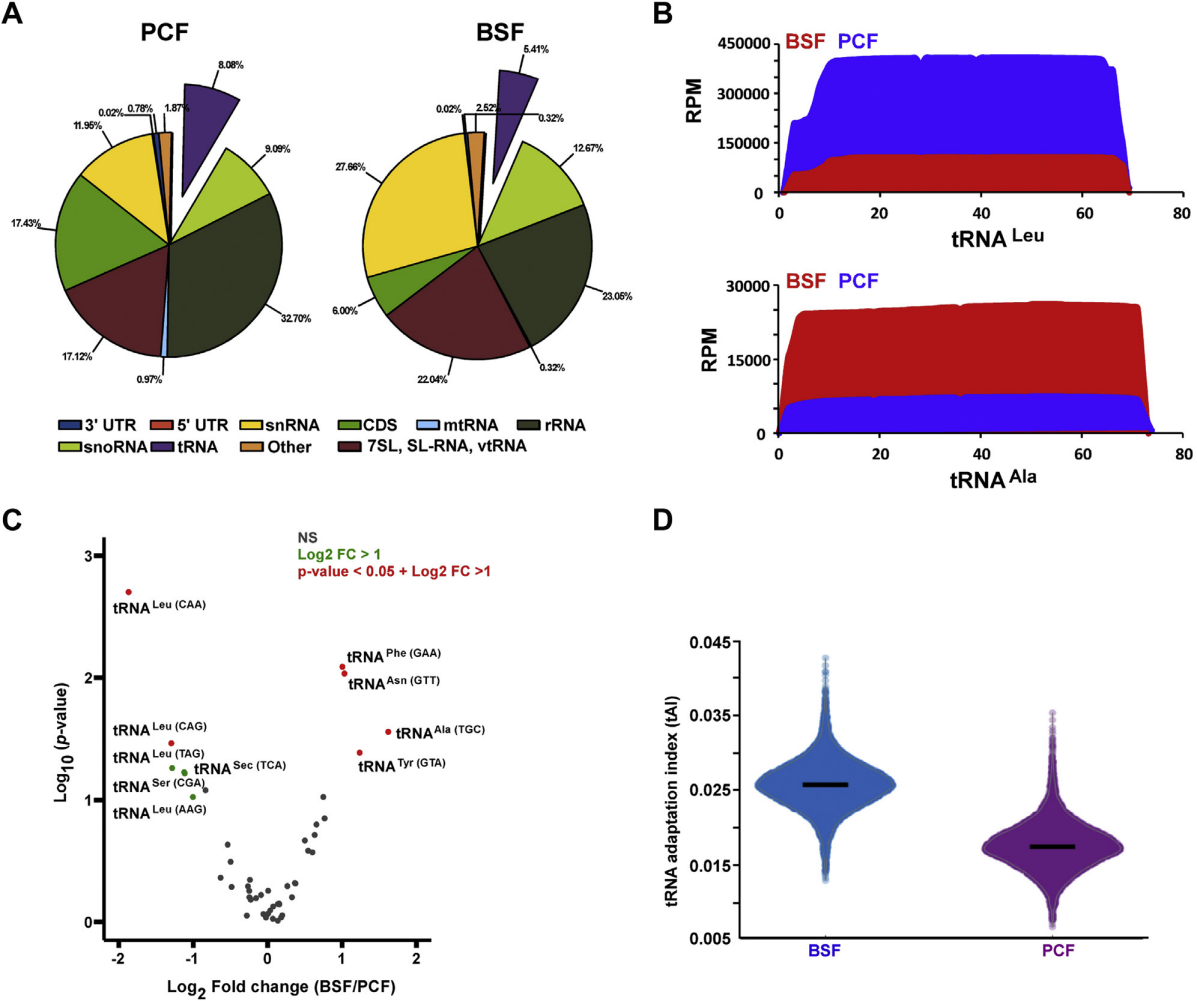
**Repertoire of tRNAs in *T. brucei*.***A*, enrichment of tRNAs in small RNA libraries prepared using the TGIRT enzyme. Whole-cell extracts from 5 × 10^9^ PCF and BSF cells was prepared, depleted of ribosomes, and RNA was extracted and used to prepare small RNA libraries using TGIRT. *B*, TGIRT small RNA libraries capture *full-length* tRNAs. Representative snapshot coverage of two tRNA is shown. Distribution of the tRNA reads across the RNA is given in reads per million (RPM). *C*, tRNAs are developmentally regulated. The volcano plot was generated by DESeq2 (59) using three independent replicates of TGIRT small RNA libraries of both PCF and BSF for the tRNA population. tRNAs that are developmentally regulated (*p*-value < 0.05, and FC > 2) are indicated in *red*. *D*, tRNAs adaptation index (tAI). To calculate tAI, tRNA abundance data from three independent replicates of TGIRT small RNA libraries of both PCF and BSF were used, and the mRNA abundance level was retrieved from a previously published study (60). BSF, bloodstream form; PCF, procyclic form; TGIRT, thermostable group II intron reverse transcriptase.

It was previously reported that mapping of modifications using conventional reverse transcriptase on tRNAs is not feasible, because the RT terminates readily on the many modifications present on tRNAs. Since the population of expressed tRNAs is not well documented in *T. brucei*, we initially determined the repertoire of tRNA species in this organism using the RNA reads obtained from the PRS library (Fig. 5*A*) and the ARAGORN tRNA detection program (40). To this end, the thermostable group II intron reverse transcriptase (TGIRT) was used to prepare the small RNA library, since this enzyme can traverse these modifications and can generate full-length transcripts of the tRNAs (41, 42) (Fig. 5*A*). The TGIRT enzyme introduces a mutation when encountering a modified nucleotide (41, 42). Indeed, using TGIRT enzyme for preparation of the small RNA libraries from PRS RNA, we obtained reads covering the entire tRNA molecule (Fig. 5*B*). Our analysis identified all the 66 tRNAs that were previously described in *T. brucei* (43) (Table S5). Next, we used the reads obtained to quantify the tRNAs in PCF and BSF life stages. The data suggested that tRNAs are also developmentally regulated (Fig. 5*C*). Two tRNAs were more abundantly expressed in PCF, and four tRNAs (tRNA^Phe^, tRNA^Asn^, tRNA^Ala^, and tRNA^Try^) were more abundantly expressed in BSF (Fig. 5*C*).

Next, we examined how the pool of tRNA in each life stage is correlated with the codon usage on the mRNA, using a parameter termed tRNA adaptation index (tAI) (Fig. 5*D*). The tAI was calculated based on total number of reads obtained for each tRNA molecule and the codon usage in each life stage (44, 45). The results show higher adaptability of the tRNA pool to codon usage of BSF compared to PCF mRNAs, based on a statistical test that was developed to assess translational selection with codon usage (44, 45).

**Figure 6:**
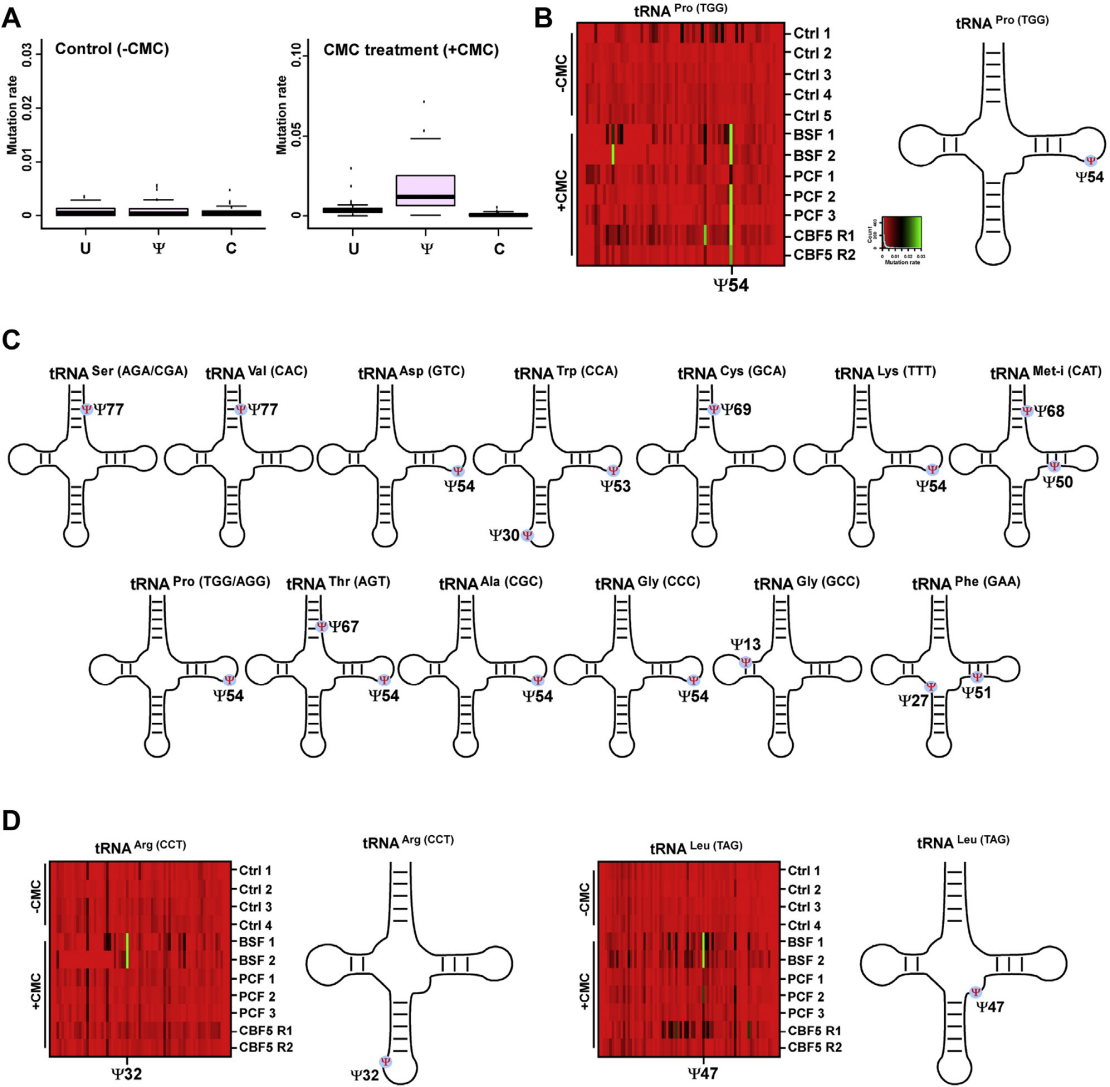
**Genome-wide detection of Ψs on tRNAs by tRNA-Ψ-seq.***A*, validation of tRNA-Ψ-seq using the universally conserved TΨC position. Mutation profile was generated for the tRNA TΨC position using both CMC treated (+CMC) and untreated (-CMC) libraries. *B*, mutation profile of tRNA^Pro (TGG)^. The mutation rate across the complete tRNA sequence is indicated by color from *red* to *green* (*low to high*) in the cell lines analyzed. CBF5: *cbf5* silenced. The Ψ position is shown on the secondary structure of tRNA. *C*, the location of Ψ indicated on the secondary structure of 15 tRNAs. *D*, Ψ on tRNAs are developmentally regulated. The mutation profiles of tRNA^Arg(CCT)^ and tRNA^Leu(TAG)^ are shown. The differentially modified Ψ position is shown at its location on the secondary structure of tRNA.

The small RNA-Seq using TGIRT was also used to identify Ψs in tRNAs. Recently, it was demonstrated that TGIRT introduces mutations in Ψ sites when treated with CMC (46). As a positive control, we assessed the quantity of mutations in the universally conserved TΨC loop of tRNAs (Fig. 6*A*). Our results (Fig. 6, *A* and *B*) suggest that the rate of mutation introduced at the Ψ site within the TΨC is higher in RNA treated with CMC (+CMC) compared to untreated RNA (-CMC). Using this method, we detected Ψ sites in 18 tRNAs (Fig. 6, *B*–*D*). Interestingly on four tRNAs, we detected more than a single Ψ, as was previously reported in humans (46) (Fig. 6*C*). The Ψ sites on tRNAs were not reduced upon *cbf5* silencing (Fig. 6, *B* and *D*), suggesting that these Ψ sites are guided by PUS enzymes. Indeed, our recent study showed that PUS1, 3, and 7 guide Ψ on tRNAs, as in yeast and mammals (24). Finally, we also quantified the rate of mutation in each Ψ site in both PCF and BSF tRNAs (Fig. 6*D*). The Ψ sites in Ψ47 in tRNA^Leu(TAG)^ and Ψ54 in tRNA^Asp(GTC)^ were hypermodified in the BSF, and Ψ32 in tRNA^Arg(CCT)^ was detected only in BSF (Fig. 6*D*), suggesting that some Ψ sites on tRNA are developmentally regulated.

## Discussion

We previously showed that trypanosome U snRNAs contain the highest number of Ψs in nature, and these are necessary for strengthening the RNA–RNA interactions and protein binding at elevated temperature (23). Here, we extended the mapping to other important ncRNA classes by using both small RNA Ψ-seq (23) and HydraPsiSeq (31). We showed that 7SL RNA, U3 snoRNA, and vtRNA also contain Ψs, and the sites are present in critical functional domains. Moreover, Ψs are found on selected C/D and H/ACA snoRNAs. Ψ modification on the C/D snoRNA is essential for optimal guiding of Nm on its rRNA target. Differential levels of Ψ on these RNAs were found in the two life stages of the parasite. In addition, mapping tRNA levels in the two life stages showed that the level of different tRNAs is developmentally regulated. The adaptability of the tRNA pool to codon usage on mRNA was also examined and found to be higher in BSF compared to PCF, which is consistent with the higher growth rate of BSF requiring more efficient translation. Ψs were mapped on tRNAs, and unique stage modifications were observed in BSF, suggesting that Ψ modification on tRNAs can also potentially fine tune translation during cycling of the parasite between its two hosts.

Our study is the first to compare the Ψs on small RNAs using both Ψ-seq and HydraPsiSeq. The location of most of the Ψ modifications were found in both methods. This discrepancy may reflect the presence of nucleotides that are modified by more than a single RNA modification or because the HydraPsiSeq requires deep coverage of sequence to identify the Ψ nucleotide, and these were not available for less abundant ncRNAs. In addition, our library preparation selects for longer fragments (>30 nt) and may miss fragments coming from the termini of small RNAs.

The Ψs detected in this study were found in RNA–RNA interaction domains or in potential protein-interaction domains. On 7SL RNA, two Ψs were found near the 5′ end of the molecule in a domain likely to interact with sRNA-76, the tRNA-like molecule that was proposed to replace the missing Alu domain of the *T. brucei* 7SL RNA (25, 26, 27). Two Ψs were found in the SRP54-binding domain, and the rest of the modifications were adjacent to SRP19 and SRP68/72, as indicated in Figure 2*A*iii (25, 26, 27). The Ψs on U3 snoRNA were also found near RNA-binding domains of proteins such as NOP1 and NOP56 (47) (Fig. 2*B*iii). In addition, Ψs are present within the interaction domain between U3 and 5′ external transcribed spacer, which was suggested based on psoralen UV crosslinking between the pre-rRNA and U3 (Fig. S3) (35). The presence of the many Ψs may strengthen the interaction domain with the pre-rRNA, which is disrupted by several mismatches as illustrated in Fig. S3. Very little is known about the proteins that bind vtRNA, and hence, the significance of the presence of this Ψ is currently unknown (28). However, the Ψ at this position may indicate its significance for the function of vtRNA (28).

The presence of Ψ in C/D interaction domains was previously observed in yeast (48). Here, we examined the location of this modification on both C/D and H/ACA snoRNAs and examined whether Ψ assists in guiding the Nm modification on the rRNA target. Whereas some of the Ψs on C/D snoRNAs were mapped to the interaction domain of the snoRNA with its target, the Ψs on H/ACA were located above the pseudouridylation pocket. These Ψs are likely to affect the binding of CBF5 and NOP10 proteins, which were shown to bind to this region of the molecule (49). These modifications may affect the structure of H/ACA, which changes when such snoRNAs guide modification on more than a single substrate. We recently showed that TB11Cs6H1, which guides on both rRNA and U2 snRNA, opens an alternative pocket upon binding of MTAP. Despite the presence of two mismatches in the interaction domain, the modification on U2 snRNA by this snoRNA is efficient (23). It is possible that Ψs on H/ACA assists in guiding modifications on ncRNAs or even on mRNAs. It was surprising to find that the modification on H/ACA (with the exception of SLA1, TB9Cs1ppH1 [Ψ21]) was not reduced following *mtap* silencing, suggesting that these modifications are likely guided by PUS enzymes (Table 1). PUS enzymes were shown to guide Ψ on yeast snRNA (50), so it is not surprising to find PUS guiding on ncRNAs in other eukaryotes as well.

Here, we show that the efficiency of C/D snoRNA guiding Nm is assisted by Ψ addition, most likely by strengthening RNA–protein interactions. Thus, Ψ level may also add a novel level of regulation, contributing to controlling the level of Nm modification on rRNA. It was demonstrated in yeast that only Ψ35, Ψ42, and Ψ44 are detected in U2 under normal conditions, but nutrient-deprivation leads to additional Ψs at positions 56 and 93, and the Ψ at position 56 can also be induced by heat shock, compromising splicing (7). In addition, the level of small Cajal body-specific RNA 1 alters the amount of Ψ on snRNA, affecting alternative splicing and embryonic development (51). Thus, changes in modification on ncRNAs could influence the function of the RNP, affecting protein translocation to the endoplasmic reticulum mediated by SRP, rRNA processing orchestrated by U3 and other snoRNAs, and splicing carried out by snRNAs.

The repertoire of *T. brucei* tRNAs was never fully described at the RNA level. However, DNA sequence analysis of the trypanosomatid genome identified 83, 66, and 120 genes in *Leishmania major*, *T. brucei*, and *Trypanosoma cruzi*, respectively (43). Our analysis (Table S5) showed that all the tRNA genes present in the genome were identified in our small RNA libraries. Interestingly, we identified tRNA genes that are preferentially expressed either in PCF and BSF. Since the transcription of tRNA genes is driven by intragenic promoters, known as the the A and B boxes, the identity of the boxes was examined in tRNAs with preferential expression in the PCF or BSF (Fig. S5) (43). The preferentially expressed PCF tRNAs (indicated in Fig. 5*C*) seem to be more closely related to class-I tRNAs (tRNA Leu^CAA^, Leu^CAG^), while the BSF preferentially expressed tRNAs (Phe^GAA^, Asn^GTT^, Ala^TGC^, Tyr^GTA^) are more similar to class-II (Fig. S5) (43). Class-I tRNAs have a short variable loop, whereas class-II tRNAs possess a longer variable loop (43). Most *T. brucei* tRNAs belong class-I, but the BSF preferentially expressed tRNA are more closely related to class II tRNA species.

Most striking was the observation of high adaptability of the tRNA pool to the codon usage on the mRNA of BSF compared to the PCF, suggesting that translation in BSF might be more efficient because of the high tRNA adaptability. Indeed, BSF parasites grow faster than PCF (52) and adaptability of tRNAs was observed in fast growing bacterial species (53). Finally, this study highlights additional levels of regulation by Ψ modification on ncRNA that may help fine-tune a variety of different RNPs involved in complex biological functions, from pre-rRNA processing to translation and protein translocation.

## Experimental procedures

### Cell growth and transfections

PCF *T. brucei*, strain 29 to 13 (54), which carries integrated genes for the T7 polymerase and the tetracycline repressor, was grown in SDM-79 medium supplemented with 10% fetal calf serum, in the presence of 50 μg/ml hygromycin. Cells were grown in the presence of 15 μg/ml G418 for generating the RNAi-silenced cell lines. The BSF of *T. brucei* 427 (cell line 1313-514) was aerobically cultivated at 37 °C under 5% CO_2_ in HMI-9 medium supplemented with 10% fetal calf serum, 2 μg/ml G418, and 2.4 μg/ml phleomycin (55).

### Generation of transgenic parasites

Procyclic cells carrying *cbf5* and *mtap* RNAi construct were derived from our previous studies (23, 33). Briefly, for silencing *cbf5* and *mtap* mRNAs, we generated stem-loop RNAi constructs carrying ∼500 nt of the genes. These constructs were integrated to the non-transcribed rDNA locus. Stem-loop RNAi constructs were linearized by *EcorV* and transfected into PC 29-13 cell line (54). Transgenic parasites were cloned by serial dilution.

### Primer extension

Primer extension was performed as previously described (22, 33). The extension products were analyzed on 10% denaturing acrylamide gels. Oligo used for primer extension is listed in Table S1.

### Preparation of small RNome

Whole-cell extracts were prepared from 10^9^ cells; after extraction with 0.3 M KCl, the ribosomes were removed by centrifugation for 3 h at 35,000 rpm in a Beckman 70.1Ti rotor (150000*g*) (Fig. 1*A*). RNA extracted from the PRS was used for library preparation, essentially as described (21).

### Hydrazine and aniline treatment

PRS RNA (5 μg) was treated with 50% hydrazine (Sigma) for 45 min (or a combination of 30, 45, and 60 min) on ice, and ethanol was precipitated (31). The RNA pellet was then resuspended in 1 M aniline (Sigma) (pH 4.5 adjusted using glacial acetic acid) until the white pellet was completely dissolved, boiled for 10 min (or a combination of 5, 10, and 15 min) at 60 °C in the dark, and immediately placed on ice. The fragmented RNA was recovered by ethanol precipitation and used for library preparation (19).

### HydraPsiSeq library preparation

To perform HydraPsiSeq, the fragmented RNA (∼800 ng) was dephosphorylated with FastAP thermosensitive alkaline phosphatase (Thermo Scientific), cleaned by Agencourt RNA clean XP beads (Beckman Coulter), and ligated to a 3′ linker using high concentration T4 RNA Ligase 1 (NEB) in a buffer containing dimethyl sulfoxide, ATP, PEG 8000, and RNase inhibitor (NEB). The ligated RNA was cleaned from excess linker using Dynabeads MyOne SILANE beads (Thermo Scientific), and first strand complementary DNA (cDNA) was prepared using the AffinityScript Reverse Transcriptase (Agilent). The RNA was subsequently degraded using 2 μl of 1 M NaOH, and the cDNA was cleaned using Dynabeads MyOne SILANE beads. The cDNA was further ligated to a 3′ adapter using a high concentration T4 RNA Ligase 1 (NEB) and cleaned of excess adapter using Dynabeads MyOne SILANE beads (Thermo Scientific). The adapter-ligated cDNA was PCR enriched using NEBNext high-fidelity (NEB) polymerase (9 PCR cycles), separated on an E-Gel EX agarose gel (Invitrogen), and size selected at the range of 150 to 300 bp (containing ∼30–180 nt corresponding to RNA). The amplicons were gel purified using NucleoSpin Gel and PCR Clean-up kit (Macherey-Nagel) and sequenced in a Nextseq system (Illumina) in paired end mode (20 million reads for each sample).

### HydraPsiSeq data analysis

The paired end reads obtained from each sample were aligned to the *T. brucei* small RNA using Smalt v_0.7.5 (http://www.sanger.ac.uk/resources/software/SMALT/) with default parameters. For each sample, the resulting bam file was sorted and filtered for proper pairs using Samtools v1.9 (56) and then converted to a BED file using the bamtobed module from the BEDtools v2.26.0 suite (57). Using an in-house Perl script on each BED file, the number of reads whose 5′-end alignments initiate at that base for each position on the rRNA was calculated. The total coverage for each base was then determined using the genomecov module from the BEDtools v2.26.0 suite (57). These files were then used as input for the R scripts (https://github.com/FlorianPichot/HydraPsiSeqPipeline), as previously described (31).

### Alkaline hydrolysis and RiboMeth-seq library preparation

Total RNA (5–10 μg) was denatured at 90 °C for 2 min in a thermocycler. Then, an equal volume of buffer (NaHCO_3_/Na_2_CO_3_, pH 9.9) was added, and RNA samples were incubated at 90 °C for 20 min (19, 39). The hydrolyzed RNA was then used for library preparation. Briefly, 800 ng of the RNA was dephosphorylated with FastAP thermosensitive alkaline phosphatase and cleaned by Agencourt RNA clean XP beads (Beckman Coulter). The RNA was then ligated to 3′ linker using high concentration T4 RNA Ligase 1 (NEB) in a buffer containing dimethyl sulfoxide, ATP, PEG 8000, and RNase inhibitor (NEB) for 1.5 h at 22 °C. The ligated RNA was purified from excess linker using Dynabeads MyOne SILANE beads, and first strand cDNA was prepared using the AffinityScript Reverse Transcriptase enzyme at 55 °C for 45 min. Next, the RNA was degraded using 2 μl of 1M NaOH, and the cDNA was cleaned using Dynabeads MyOne SILANE beads. The cDNA was further ligated to 3′ adapter using a high concentration T4 RNA Ligase 1 (NEB) overnight at 22 °C and cleaned of excess adapter by using Dynabeads MyOne SILANE beads. The adapter ligated cDNA was PCR enriched using NEBNext high-fidelity polymerase (9 PCR cycles), separated on an E-Gel EX agarose gel, and size selected for the range of 150 to 300 bp (containing ∼30–180 nt corresponding to RNA). The amplicons were gel purified using NucleoSpin Gel and PCR Clean-up kit and sequenced in a Nextseq system in paired end mode (20–40 million reads for each sample).

### RiboMeth-seq data analysis

To analyze the RiboMeth-seq libraries, the reads were initially trimmed of adapter sequences using Trim Galore version 0.4.4 (https://github.com/FelixKrueger/TrimGalore) with the following parameters:--stringency 4 --length 30 --paired --retain_unpaired. The alignment to the reference rRNA sequence was done by STAR (ver 2.0.6) (https://github.com/alexdobin/STAR/releases) (58), mapped, and properly paired reads were converted to ∗.bed using BEDtools v2.26.0 suite (57). The 5′- and 3′-ends counting was done directly on ∗.bed file using a dedicated Unix script. Analysis was performed by calculation of MAX score for detection of Nm residues and relative methylation score (score C) for their quantification. To calculate MAX score, the relative change of end coverage position by position was calculated in 5′→3′ and reverse direction. The relative change was normalized to average values spanning -6 and +6 nucleotides. The normalized relative change for 5′→3′ and reverse direction were averaged, and the maximal value between the average and normalized relative change was retained (score MAX). Relative methylation score was calculated essentially as described previously for score C using the same relative impact of neighboring nucleotides (39).

### Calculation of tAI

tAI was calculated for each mRNA in the two life stages, similarly to the tAI measure of translation efficiency (44). Here, the w-values for each codon were calculated using the normalized number of reads aligned to the tRNA genes, while considering fully matched tRNAs and tRNAs that translate through wobble roles. Then, the tAI was determined for each mRNA, by calculating the geometric mean of all w-values in the power of the codon frequency.

## Data availability

The small RNA HydraPsiSeq sequencing data related to this study have been deposited in the NCBI BioProject database under the accession number PRJNA797695 (https://eur02.safelinks.protection.outlook.com/?url=https%3A%2F%2Fdataview.ncbi.nlm.nih.gov%2Fobject%2FPRJNA797695%3Freviewer%3Dpbla7s5ift4qbl8i31f0uj1vm8&data=04%7C01%7Ctirza.doniger%40biu.ac.il%7C67e6e9b0f3324592855b08d9d99dd61f%7C61234e145b874b67ac198feaa8ba8f12%7C1%7C0%7C637780094192200189%7CUnknown%7CTWFpbGZsb3d8eyJWIjoiMC4wLjAwMDAiLCJQIjoiV2luMzIiLCJBTiI6Ik1haWwiLCJXVCI6Mn0%3D%7C3000&sdata=FiPh%2Bb3y1nefbTQbECYgLO7DnOm7B76xKT3EOSamTag%3D&reserved=0). The RiboMeth-seq data is available under the accession number PRJNA836748, PRJNA526606, and PRJNA776556. The small RNA Ψ-seq (23), RNA interactome (32) were derived from our previous study and were deposited under the accession number PRJNA476671 and PRJNA630014, respectively.

## Code availability

All bioinformatics scripts used in this study are available from the corresponding author upon request. Scripts used to analyze HydraPsiSeq and RiboMeth-seq are available at https://github.com/michaelilab/TB_Pseudo_small_ncRNA.

## Supporting information

This article contains supporting information (28, 35, 43).

## Conflict of interest

The authors declare that they have no conflicts of interest with the contents of this article.

## Supporting information

Supplementary Figure and Table T1–T4

Supplementary_Table_T5
