# Supplementary material for: Identification and functional implications of pseudouridine RNA modification on small noncoding RNAs in the mammalian pathogen Trypanosoma brucei
Source: J Biol Chem. 2022 Jun 14;298(7):102141. doi: 10.1016/j.jbc.2022.102141 (PMC9283944; doi:10.1016/j.jbc.2022.102141)
Supplement: Supplementary Figure and Table T1–T4 [file mmc1.docx]

**SUPPLEMENTARY FIGURE LEGENDS**

**Figure S1. Ψ-seq analysis of on vtRNA. (A) Ψ on vtRNA is guided by H/ACA snoRNA.** Ψ-fc(log2) values (y-axis) were determined for both *cbf5* -TET and +TET based on small RNA Ψ-seq libraries. Representative line graph of vtRNA is presented. Three independent biological replicates of small RNA Ψ-seq were used to validate the sensitivity of *T. brucei* vtRNA Ψ to *cbf* silencing. **(B) Ψ on vtRNA is guided by H/ACA snoRNA associated with MTAP.** Ψ-fc(log2) values (y-axis) were determined for both *mtap* -TET and +TET based on small RNA Ψ-seq libraries. Representative line graph of vtRNA is presented. Three independent biological replicates of small RNA Ψ-seq were used to validate the sensitivity of *T. brucei* vtRNA Ψ to *mtap* silencing.


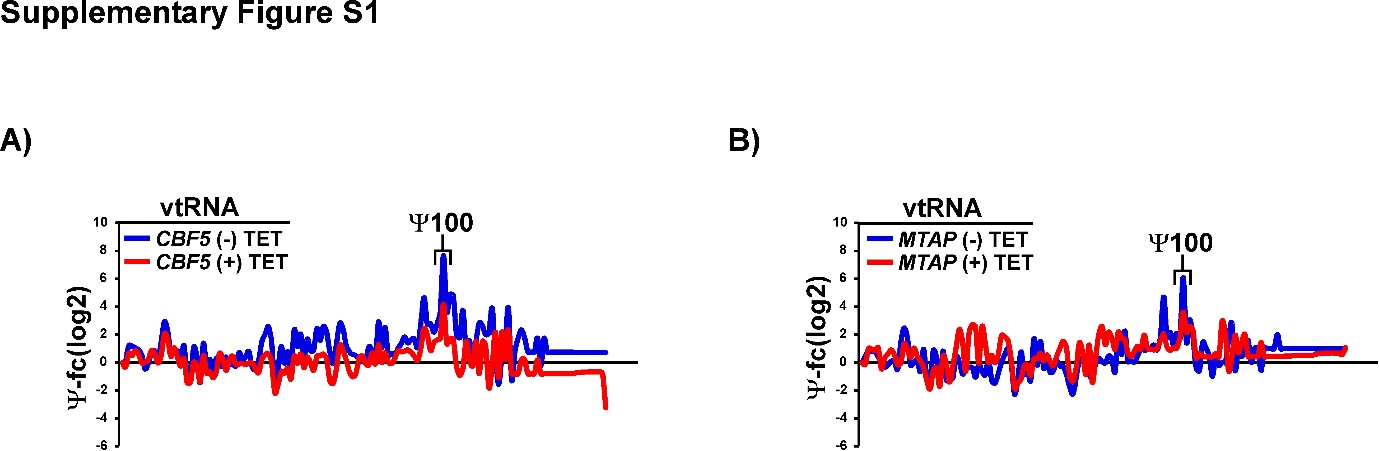


**Figure S2. Potential base pairing of H/ACA snoRNA with target RNA to guide novel Ψs** **detected in this study.**


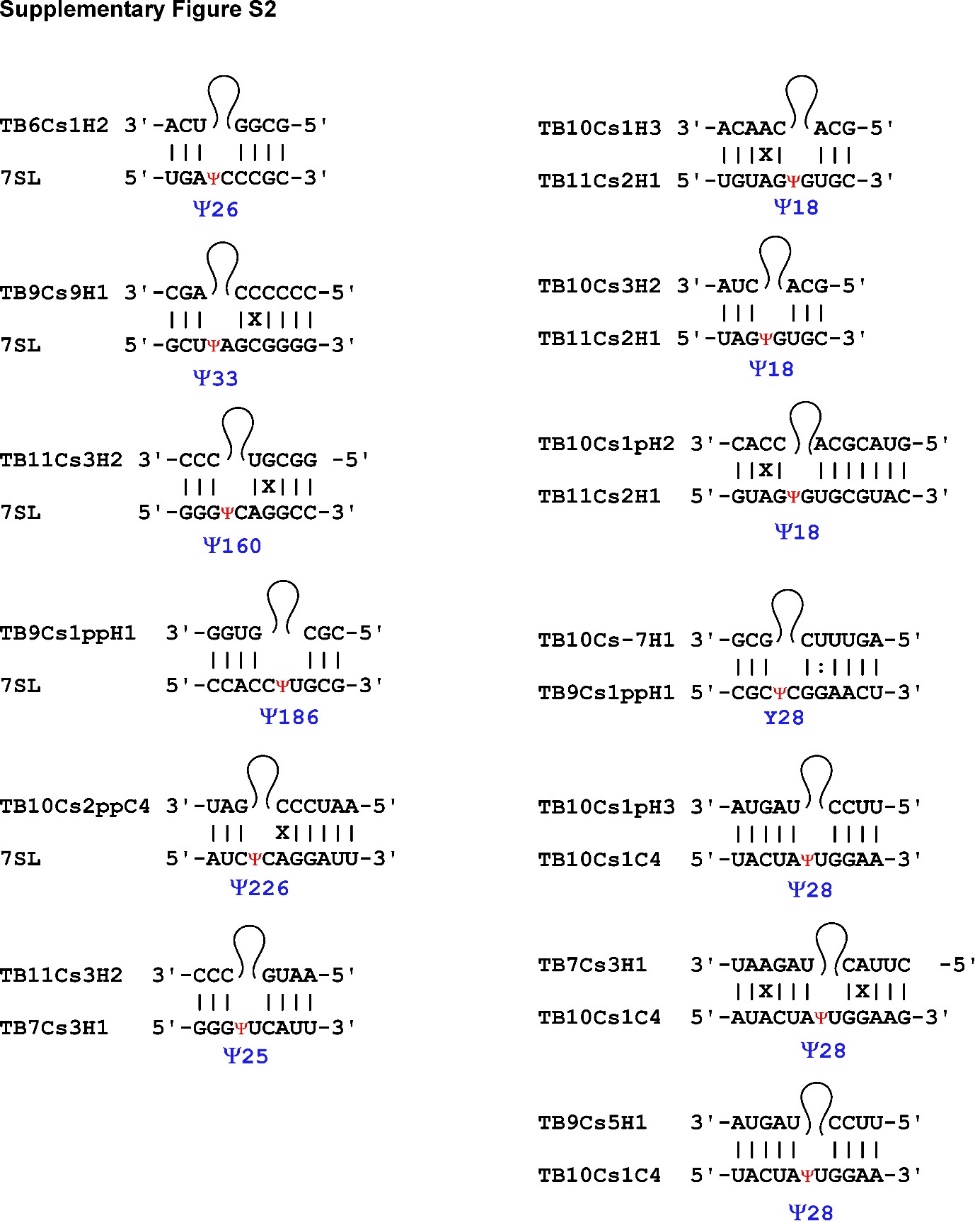


**Figure S3. Ψs in U3 are located in the interaction domain with the 5’external transcribed spacer (ETS) of the pre-RNA** (35)**.**


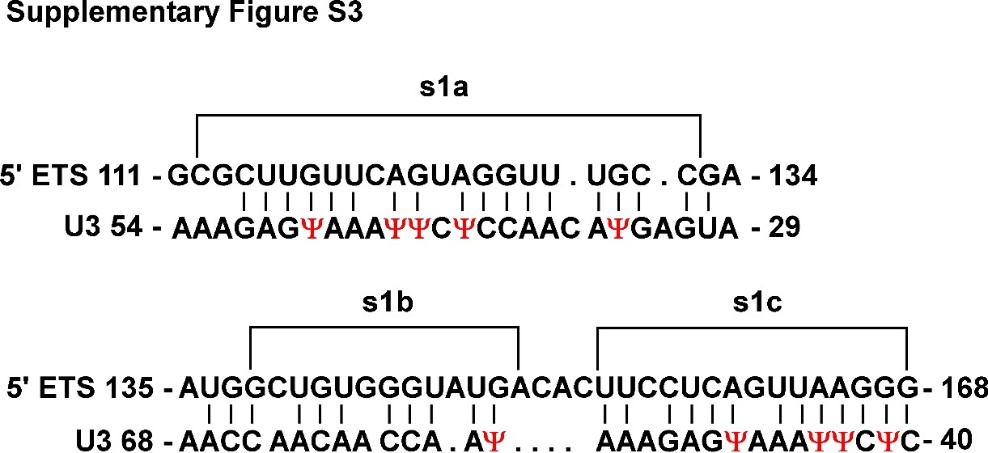


**Figure S4. Ψ-seq analysis of on *T. brucei* C/D snoRNAs. (i) Detection of Ψs on C/D snoRNA**. Ψ-fc(log2) values (y-axis) were determined for both PCF and BSF small RNA Ψ-seq libraries. Representative line graph of 9 C/D snoRNAs are presented. **(ii) Localization of Ψs on C/D snoRNA.** Scheme depicting the position of Ψs on the secondary structure of C/D snoRNA highlighting C/C’ and D/D’ boxes, and the potential base pairing with the rRNA target is indicated.


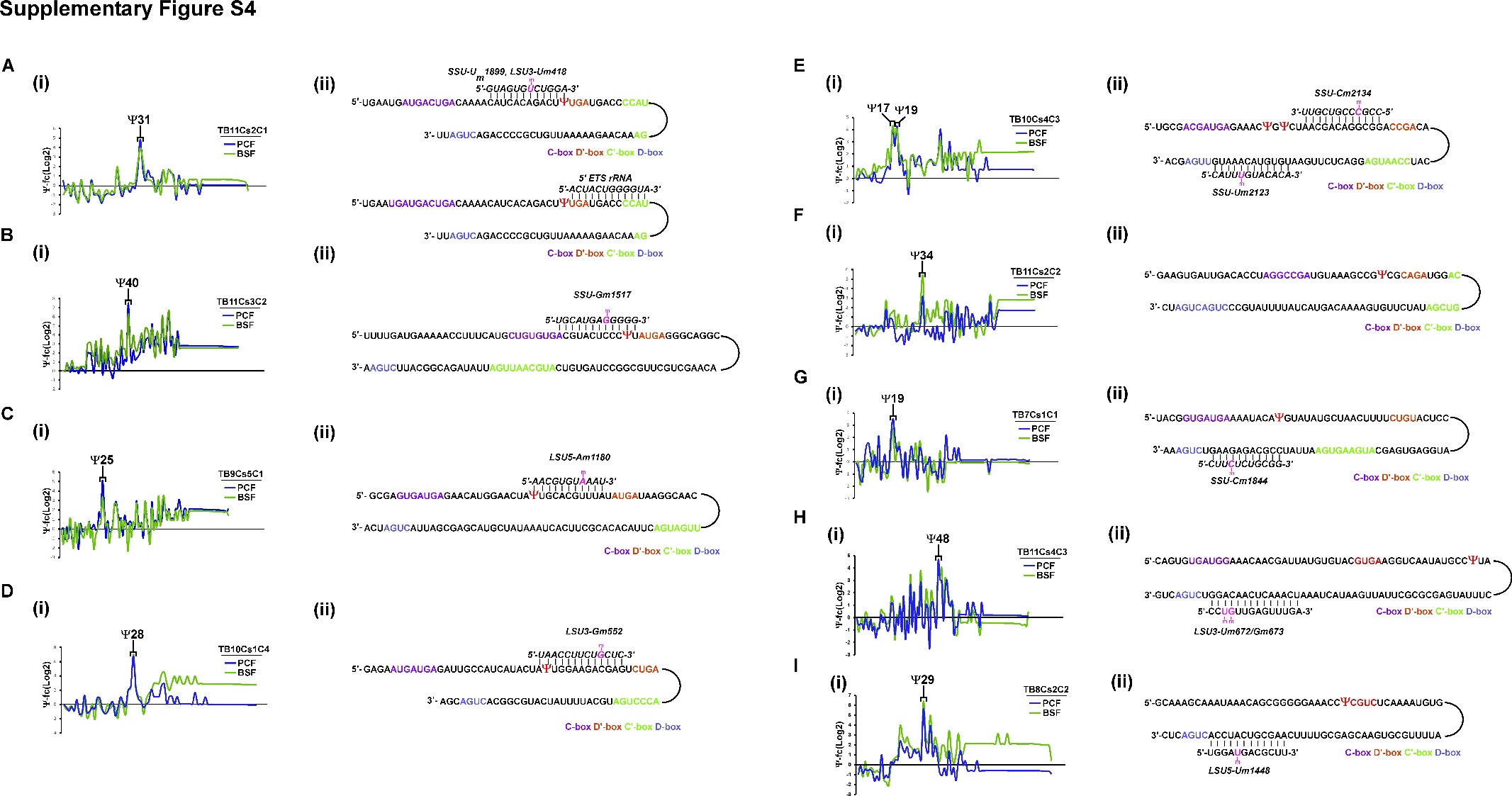


**Figure S5. Multiple sequence alignment of *T. brucei* tRNA sequences. The** TCOFFEE multiple sequence alignment program was used to align the sequences (<https://tcoffee.crg.eu/>). The A and B boxes are indicated by red and blue rectangles, respectively. The identity of boxes A and B was derived from (43).


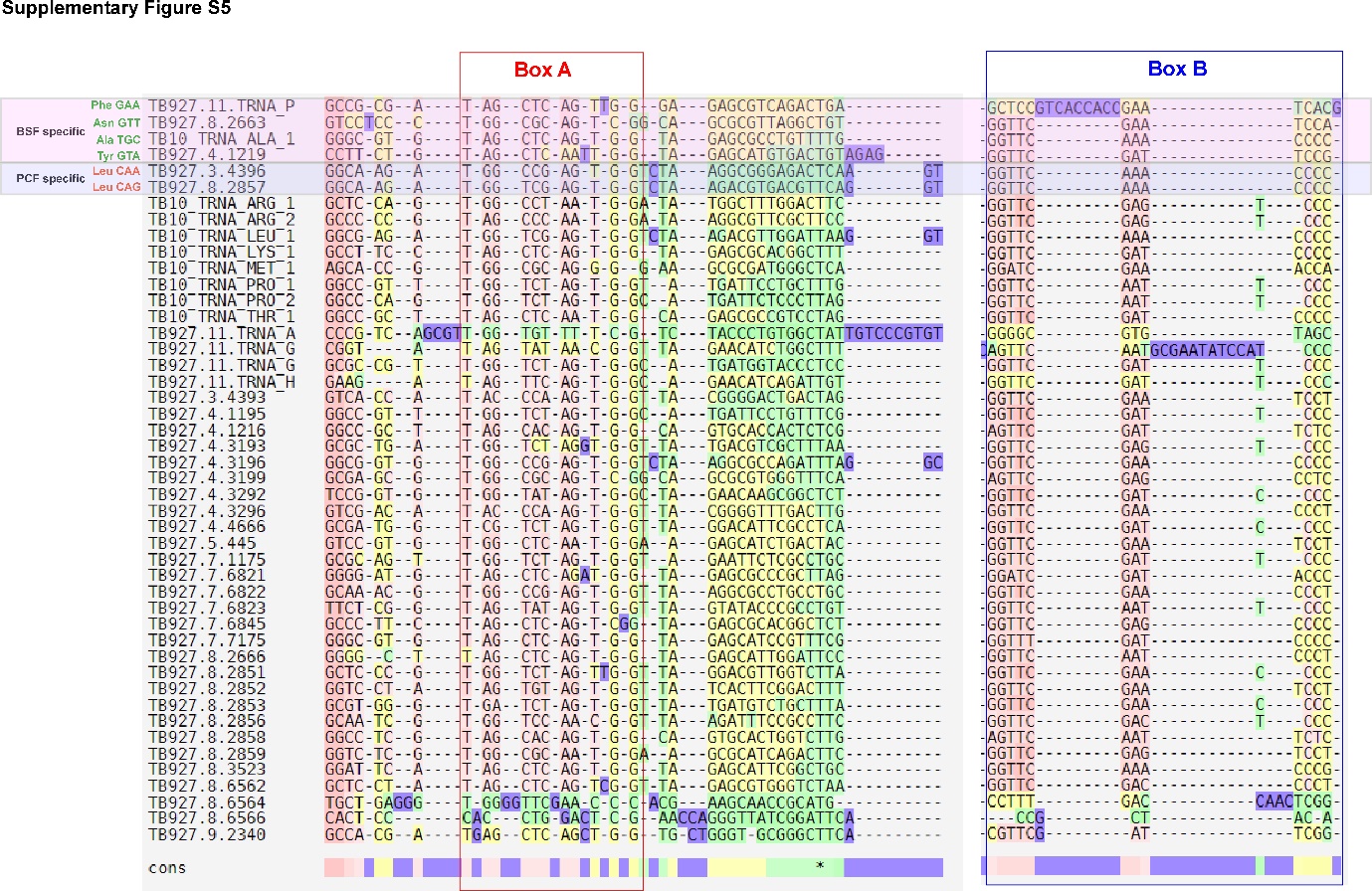


**SUPPLEMENTARY TABLE LEGENDS**

**Supplementary Table T1. List of primers used in this study.**

Primer used for primer extension:

U3_R_Pseudo GGATCCTTCTGGAACCGGCT

**Supplementary Table T2. List of H/ACA snoRNAs crosslinked to 7SL RNA.** Data are presented as total number of reads obtained from small RNA interactome libraries. Lig (-): Ligation minus; Lig (+): ligation plus small RNA interactome libraries.

|  | snoRNA | Type | Lig - R1 | Lig – R2 | UV – Lig + R1 | UV - Lig + R2 | UV - Lig + R3 | UV + Lig + R1 | UV + Lig + R2 | UV + Lig + R3 | UV + Lig + R4 |
| --- | --- | --- | --- | --- | --- | --- | --- | --- | --- | --- | --- |
| 7SL | TB10Cs1H1 | H/ACA | 0 | 0 | 3 | 32 | 11 | 0 | 0 | 1 | 0 |
| 7SL | TB10Cs1H2 | H/ACA | 0 | 0 | 2 | 28 | 1 | 1 | 0 | 0 | 0 |
| 7SL | TB10Cs1H3 | H/ACA | 0 | 0 | 1 | 40 | 16 | 1 | 0 | 2 | 0 |
| 7SL | TB10Cs1pH1 | H/ACA | 0 | 0 | 0 | 3 | 0 | 0 | 0 | 0 | 0 |
| 7SL | TB10Cs1pH2 | H/ACA | 0 | 0 | 3 | 67 | 4 | 2 | 2 | 4 | 0 |
| 7SL | TB10Cs3H1 | H/ACA | 0 | 0 | 0 | 14 | 11 | 6 | 2 | 4 | 0 |
| 7SL | TB10Cs3H2 | H/ACA | 0 | 0 | 2 | 12 | 5 | 2 | 1 | 0 | 0 |
| 7SL | TB10Cs4H2 | H/ACA | 0 | 0 | 0 | 1 | 1 | 0 | 0 | 0 | 0 |
| 7SL | TB10Cs4H3 | H/ACA | 0 | 0 | 2 | 4 | 1 | 4 | 0 | 0 | 0 |
| 7SL | TB10Cs4H4 | H/ACA | 0 | 0 | 0 | 14 | 5 | 0 | 1 | 3 | 0 |
| 7SL | TB10Cs5H2 | H/ACA | 0 | 0 | 0 | 1 | 0 | 0 | 0 | 0 | 0 |
| 7SL | TB10Cs5H3 | H/ACA | 0 | 0 | 0 | 3 | 0 | 0 | 0 | 0 | 0 |
| 7SL | TB10Cs6ppH1 | H/ACA | 0 | 0 | 0 | 2 | 0 | 0 | 0 | 0 | 0 |
| 7SL | TB10Cs-7H1 | H/ACA | 0 | 0 | 0 | 2 | 1 | 0 | 0 | 0 | 0 |
| 7SL | TB9Cs1H1 | H/ACA | 0 | 0 | 0 | 4 | 1 | 2 | 0 | 0 | 0 |
| 7SL | TB9Cs1H2 | H/ACA | 0 | 0 | 0 | 0 | 1 | 0 | 0 | 0 | 0 |
| 7SL | TB9Cs1H3 | H/ACA | 0 | 0 | 0 | 3 | 0 | 0 | 0 | 0 | 0 |
| 7SL | TB9Cs1pH1 | H/ACA | 0 | 0 | 0 | 2 | 1 | 0 | 0 | 0 | 0 |
| 7SL | TB9Cs1ppH1 | H/ACA | 0 | 0 | 0 | 8 | 1 | 0 | 0 | 0 | 0 |
| 7SL | TB9Cs2H1 | H/ACA | 0 | 0 | 0 | 6 | 1 | 3 | 0 | 0 | 0 |
| 7SL | TB9Cs2H2 | H/ACA | 0 | 0 | 0 | 3 | 1 | 2 | 0 | 0 | 0 |
| 7SL | TB9Cs3H1 | H/ACA | 0 | 0 | 0 | 2 | 0 | 0 | 0 | 0 | 0 |
| 7SL | TB9Cs3H2 | H/ACA | 0 | 0 | 0 | 1 | 0 | 1 | 0 | 0 | 0 |
| 7SL | TB9Cs4H1 | H/ACA | 0 | 0 | 1 | 33 | 12 | 0 | 1 | 2 | 1 |
| 7SL | TB9Cs4H2 | H/ACA | 0 | 0 | 4 | 47 | 10 | 1 | 2 | 1 | 0 |
| 7SL | TB9Cs4H3 | H/ACA | 0 | 0 | 1 | 7 | 6 | 2 | 1 | 1 | 0 |
| 7SL | TB9Cs5H1 | H/ACA | 0 | 0 | 0 | 4 | 0 | 1 | 0 | 0 | 0 |
| 7SL | sRNA-76 | ncRNA | 0 | 0 | 7 | 53 | 12 | 6 | 5 | 7 | 1 |

**Supplementary Table T3. The fold-change of Ψs in PCF versus BSF based on Ψ-seq for U3 snRNAs**. Ψ-fc(log2) was calculated across three independent biological replicates for pseudouridylated sites. Ψ-fc(log2) >1.3 (BSF/PCF) with *p*<0.05 was considered as hypermodified Ψ.

| snRNA | Ψ position | BSF vs PCF Rep1(Log2FC) | BSF vs PCF Rep2(Log2FC) | BSF vs PCF Rep 3(Log2FC) | Average (Log2FC) | *p*-value | Hypermodified |
| --- | --- | --- | --- | --- | --- | --- | --- |
| U3 | 8 | 1.17 | 1.27 | 1.68 | 1.37 | 0.07 |  |
| U3 | 11 | 1.00 | 1.37 | 1.87 | 1.42 | 0.12 |  |
| U3 | 13 | 1.36 | 1.54 | 1.78 | 1.56 | 0.02 | Hypermodified |
| U3 | 22 | 1.31 | 1.58 | 1.09 | 1.32 | 0.07 |  |
| U3 | 34 | 1.29 | 1.53 | 1.30 | 1.37 | 0.02 | Hypermodified |
| U3 | 41 | 1.12 | 1.03 | 1.30 | 1.15 | 0.10 |  |
| U3 | 43 | 1.30 | 1.19 | 1.66 | 1.38 | 0.06 |  |
| U3 | 55 | 1.22 | 0.63 | 1.54 | 1.13 | 0.34 |  |
| U3 | 70 | 1.32 | 0.96 | 0.85 | 1.05 | 0.39 |  |
| U3 | 73 | 1.40 | 0.97 | 0.96 | 1.11 | 0.26 |  |
| U3 | 76 | 1.37 | 1.27 | 0.82 | 1.15 | 0.23 |  |
| U3 | 79 | 1.29 | 1.26 | 0.94 | 1.17 | 0.14 |  |
| U3 | 85 | 1.06 | 1.07 | 0.97 | 1.04 | 0.19 |  |
| U3 | 86 | 1.05 | 1.05 | 0.95 | 1.02 | 0.32 |  |
| U3 | 89 | 1.12 | 1.14 | 1.01 | 1.09 | 0.08 |  |
| U3 | 93 | 1.77 | 0.99 | 2.15 | 1.64 | 0.10 |  |
| U3 | 107 | 1.43 | 1.53 | 1.00 | 1.32 | 0.09 |  |
| U3 | 109 | 0.47 | 1.45 | 1.23 | 1.05 | 0.44 |  |
| U3 | 110 | 1.05 | 1.12 | 1.14 | 1.10 | 0.03 |  |
| U3 | 114 | 1.60 | 1.45 | 1.22 | 1.42 | 0.03 | Hypermodified |

**Supplementary Table T4. List of H/ACA snoRNAs crosslinked to U3 snoRNA.** The data are presented as total number of reads obtained from small RNA interactome libraries. Lig (-): Ligation minus, Lig (+): ligation plus small RNA interactome libraries.

| snoRNA | Type | snoRNA | Lig - R1 | Lig – R2 | UV - R1 Lig + | UV - R2 Lig + | UV - R3 Lig + | UV + R1 Lig + | UV + R2 Lig + | UV + R3 Lig + | UV + R4 Lig + |
| --- | --- | --- | --- | --- | --- | --- | --- | --- | --- | --- | --- |
| TB10Cs1H1 | H/ACA | U3 | 0 | 0 | 0 | 2 | 0 | 0 | 0 | 0 | 0 |
| TB10Cs1H2 | H/ACA | U3 | 0 | 0 | 0 | 1 | 0 | 0 | 0 | 0 | 0 |
| TB10Cs1H3 | H/ACA | U3 | 0 | 0 | 0 | 0 | 0 | 1 | 0 | 0 | 0 |
| TB10Cs1pH2 | H/ACA | U3 | 0 | 0 | 0 | 1 | 1 | 0 | 0 | 0 | 0 |
| TB10Cs3H1 | H/ACA | U3 | 0 | 0 | 0 | 2 | 0 | 0 | 0 | 0 | 0 |
| TB10Cs3H2 | H/ACA | U3 | 0 | 0 | 0 | 1 | 0 | 0 | 0 | 0 | 0 |
| TB10Cs4H4 | H/ACA | U3 | 0 | 0 | 0 | 2 | 0 | 0 | 0 | 0 | 0 |
| TB9Cs2H1 | H/ACA | U3 | 0 | 0 | 0 | 1 | 0 | 0 | 0 | 0 | 0 |
| TB9Cs3H2 | H/ACA | U3 | 0 | 0 | 0 | 0 | 1 | 1 | 1 | 0 | 0 |
| TB9Cs4H1 | H/ACA | U3 | 0 | 0 | 0 | 1 | 0 | 0 | 0 | 0 | 0 |
| TB9Cs4H2 | H/ACA | U3 | 0 | 0 | 0 | 6 | 0 | 1 | 0 | 0 | 0 |
| TB9Cs4H3 | H/ACA | U3 | 0 | 0 | 0 | 0 | 1 | 0 | 0 | 0 | 0 |
